# Astrocytic 4R tau expression drives astrocyte reactivity and dysfunction

**DOI:** 10.1172/jci.insight.152012

**Published:** 2022-01-11

**Authors:** Lubov A. Ezerskiy, Kathleen M. Schoch, Chihiro Sato, Mariana Beltcheva, Kanta Horie, Frank Rigo, Ryan Martynowicz, Celeste M. Karch, Randall J. Bateman, Timothy M. Miller

**Affiliations:** 1 Department of Neurology and; 2 Center of Regenerative Medicine, Washington University School of Medicine, St. Louis, Missouri, USA.; 3 Ionis Pharmaceuticals, Carlsbad, California, USA.; 4 Department of Psychiatry, Washington University School of Medicine, St. Louis, Missouri, USA.

**Keywords:** Neuroscience, Neurodegeneration, Neurological disorders, iPS cells

## Abstract

The protein tau and its isoforms are associated with several neurodegenerative diseases, many of which are characterized by greater deposition of the 4-repeat (4R) tau isoform; however, the role of 4R tau in disease pathogenesis remains unclear. We created antisense oligonucleotides (ASOs) that alter the ratio of 3R to 4R tau to investigate the role of specific tau isoforms in disease. Preferential expression of 4R tau in human tau–expressing (hTau-expressing) mice was previously shown to increase seizure severity and phosphorylated tau deposition without neuronal or synaptic loss. In this study, we observed strong colocalization of 4R tau within reactive astrocytes and increased expression of pan-reactive and neurotoxic genes following 3R to 4R tau splicing ASO treatment in hTau mice. Increasing 4R tau levels in primary astrocytes provoked a similar response, including a neurotoxic genetic profile and diminished homeostatic function, which was replicated in human induced pluripotent stem cell–derived (iPSC-derived) astrocytes harboring a mutation that exhibits greater 4R tau. Healthy neurons cultured with 4R tau–expressing human iPSC–derived astrocytes exhibited a higher firing frequency and hypersynchrony, which could be prevented by lowering tau expression. These findings support a potentially novel pathway by which astrocytic 4R tau mediates reactivity and dysfunction and suggest that astrocyte-targeted therapeutics against 4R tau may mitigate neurodegenerative disease progression.

## Introduction

Tauopathies are a class of neurodegenerative diseases characterized by the pathogenic aggregation of hyperphosphorylated tau protein in neurofibrillary tangles (1). Tau, encoded by the microtubule associated protein tau (*MAPT*) gene, plays a role in many critical cellular functions, including intracellular signaling, synapse formation and function, and axonal transport regulation (2–4). Tau hyperphosphorylation and aggregation can disrupt essential cellular processes critical for maintaining homeostasis in the central nervous system (5) and lead to neurodegenerative disease (1, 6, 7).

Tau can be classified as 3-repeat (3R) or 4-repeat (4R), corresponding to the number of repeat domains that result from alternative splicing of exon 10 of *MAPT* (8–10). In adults, the ratio of 3R to 4R tau is approximately 1:1; however, certain disease mutations in the *MAPT* gene can cause a change in the ratio of the isoforms toward greater expression of either 3R or 4R tau (11–13). Many primary genetic *MAPT* mutations are found in and around exon 10, resulting in tau mis-splicing, biased 4R tau production, or mutant tau protein expression (1, 14, 15). Thus, an altered ratio of tau isoforms, particularly affecting the 4R tau isoform, is sufficient to drive tau pathology. In a mouse model expressing all 6 isoforms of human tau (hTau), including both 3R and 4R tau (16), an antisense oligonucleotide (ASO) strategy to increase 4R tau alone induced tau phosphorylation and aggregation and increased seizure severity (17). These results further confirmed that 4R tau toxicity might be involved in tau pathology and disease. While there were substantial effects on neuronal function as evidenced by behavioral changes, there were minimal neuronal changes in pathology or number. This discrepancy prompted us to consider non-neuronal cells as a potential contributor to 4R tau toxicity, focusing first on astrocytes because of the well-described pathological changes within astrocytes in human disease (18–22).

To examine an astrocyte-specific role for 4R tau in disease, we used tau isoform–switching ASOs, previously characterized in our lab (17), to alter the ratio of tau isoform expression in hTau-expressing mouse and human astrocytes. Our findings demonstrate that, despite the low level of tau expression in astrocytes, 4R tau interferes with normal astrocyte functions, causing neuronal hyperexcitability, which together may contribute to neurodegeneration.

## Results

### Reactive astrocytes express 4R tau in vivo.

**Figure 1:**
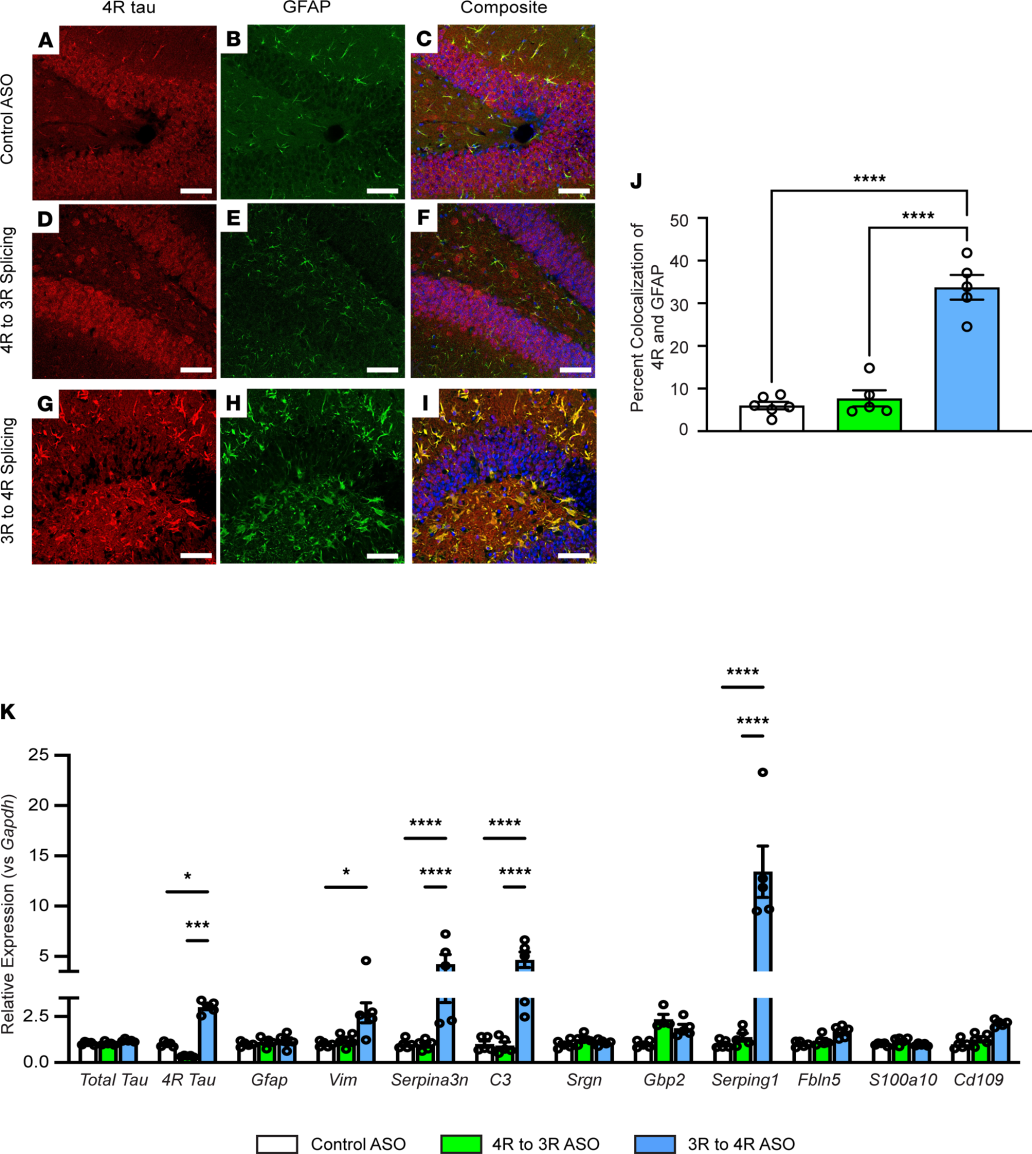
4R tau expression in astrocytes leads to a reactive phenotype in vivo. (**A**, **D**, and **G**) 4R tau, (**B**, **E**, and **H**) glial fibrillary acidic protein (GFAP), and (**C**, **F**, and **I**) merged representative images of the dentate gyrus of the hippocampus, contralateral to ASO injection, in hTau mice treated with control ASO, 4R to 3R tau splicing ASO, or 3R to 4R tau splicing ASO. Scale bar: 50 μm. (**J**) Percentage of the total visual field that had 4R tau colocalized with GFAP. Data are shown as mean ± SEM; 2-way ANOVA with Tukey’s multiple comparisons; *n* = 5–6 mice per treatment, ****P < 0.0001. (**K**) Expression of select pan-reactive (*Gfap*, *Vimentin*, and *Serpina3n*), neurotoxic (*C3*, *Srgn*, *Gbp2*, *Serping1*, and *Fbln5*), and neuroprotective (*S100a10* and *Cd109*) genes in hTau mouse brain lysates after control, 4R to 3R tau splicing, or 3R to 4R tau splicing ASO treatment measured by qRT-PCR. Data are normalized to *Gapdh* relative to control ASO levels and shown as mean ± SEM; 2-way ANOVA with Tukey’s multiple comparisons; *n* = 5–6 mice per treatment, *P < 0.05, ***P < 0.001, ****P < 0.0001.

hTau mice, which express all isoforms of hTau in a mouse tau–knockout (mTau^–/–^) background, were treated with ASOs that bias toward the inclusion of tau exon 10 (3R to 4R splicing ASO) or exclusion of exon 10 (4R to 3R splicing ASO). Despite observing changes in behavior in previous studies of 3R to 4R splicing ASO-treated hTau mice (17), we had not identified overt changes to neuronal pathology or number. Thus, to explore tau isoform localization further, we stained for 3R and 4R tau isoforms within the brain. In control ASO- and 4R to 3R splicing ASO-treated brain tissue, we observed both 3R and 4R tau to be primarily neuronal, with some 4R tau colocalizing to astrocytes (Figure 1, A–F, and Supplemental Figure 1; supplemental material available online with this article; https://doi.org/10.1172/jci.insight.152012DS1). In contrast, tissue from hTau mice treated with the 3R to 4R tau splicing ASO exhibited a change in 4R tau localization coincident with astrocyte morphology (Figure 1, G–I) not seen in the hTau mice treated with the control ASO or the 4R to 3R tau splicing ASO. We saw that treatment with the 3R to 4R tau splicing ASO significantly increased the amount of 4R tau and decreased the amount of 3R tau (Supplemental Figure 1Q). We also identified a significant increase in 4R tau and GFAP colocalization in the hTau mice that were treated with the 3R to 4R tau splicing ASO, which was not apparent in those treated with either the control ASO or the 4R to 3R tau splicing ASO (Figure 1J).

After identifying a change in astrocyte morphology, we performed quantitative real-time PCR (qRT-PCR) analysis for select pan-reactive, neurotoxic, and neuroprotective genes from whole mouse brain lysates from hTau, mTau^–/–^, and wild-type (C57BL/6) mice treated with either control or 3R to 4R tau splicing ASO. hTau mice treated with the 3R to 4R tau splicing ASO exhibited a significant upregulation of mRNA of several reactive and neurotoxic genes (23), including *Vim* (pan-reactive), *Serpina3n* (pan-reactive), *C3* (neurotoxic), and *Serping1* (neurotoxic) compared with the mice treated with the control ASO and the 4R to 3R tau splicing ASO (Figure 1K). No change in neuroprotective gene expression was identified in the hTau mice treated with the 3R to 4R tau splicing ASO compared to those treated with the control ASO or the 4R to 3R tau splicing ASO (Figure 1K). No effects on the expression of these genes were evident in either mTau^–/–^ or C57BL/6 mice treated with the same ASOs (Supplemental Figure 2, A and B), showing that the mRNA changes after 3R to 4R ASO treatment were specific to altering the ratio of 4R tau and not an inflammatory, nonspecific effect of ASOs.

**Figure 2:**
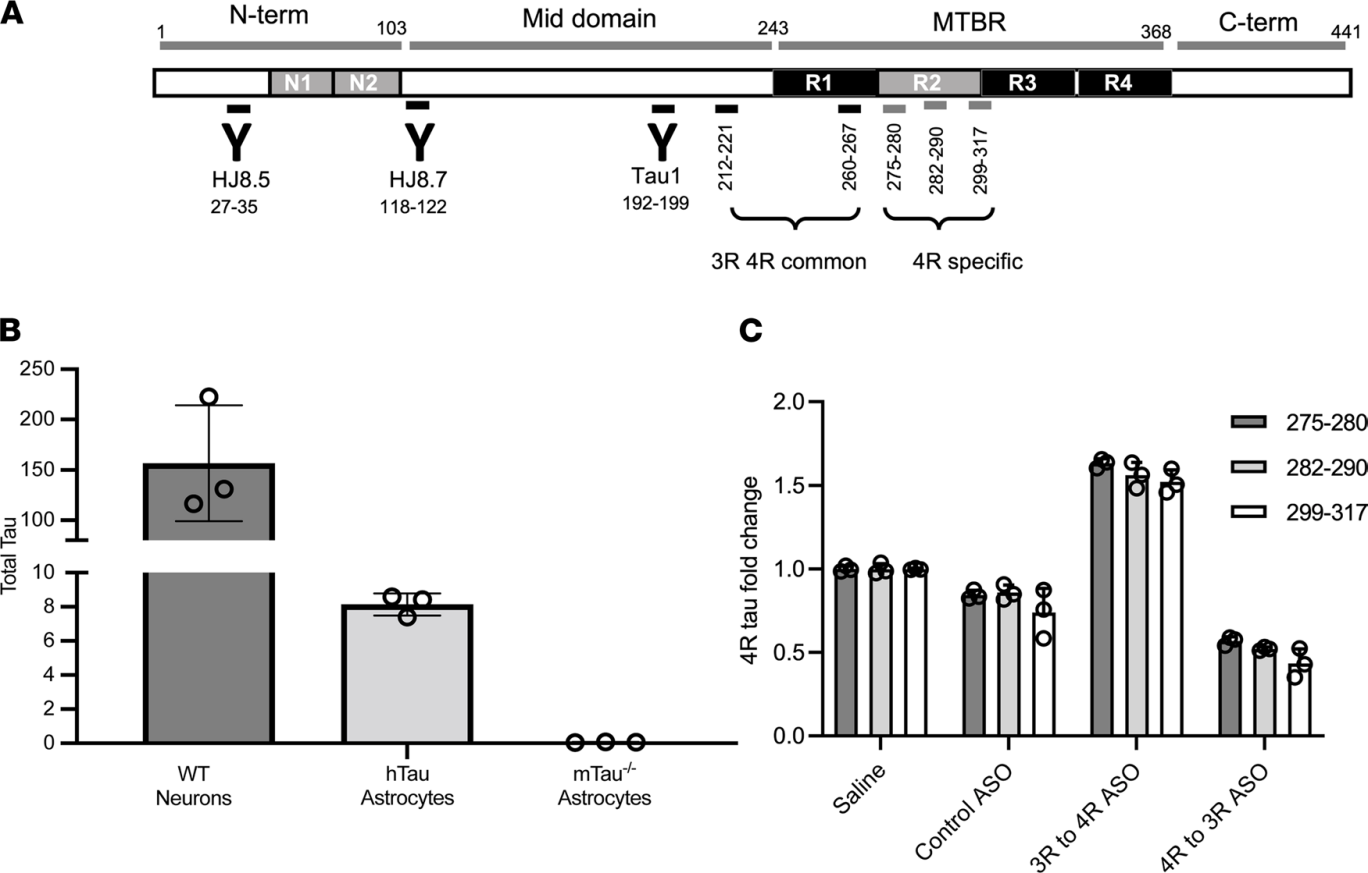
Tau and isoform expressions at protein level measured by mass spectrometry. (**A**) Schematics of tau protein, antibody epitopes, and peptides used for mass spectrometry analyses. MTBR, microtubule binding region. (**B**) hTau astrocytes have approximately 100-fold less tau expression as compared with WT neurons measured by 212-221 mid domain peptide (“Total tau”) that is common to all isoforms, *n* = 3 biological replicates/group. (**C**) Three 4R isoform-specific peptides — (a) 282-290, (b) 275-280, (c) 299-317 — that are in R2 region and 1 common peptide (260-267) that is in proximity were measured by quantitative mass spectrometry in ASO-treated hTau astrocytes. The ratio of each 4R-specific peptide to common peptide was calculated in astrocytes (282-290/260-267, 275-280/260-267, 299-317/260-267) and compared with the ratio of saline. 3R to 4R ASO increased 4R-specific peptides 1.53 ± 0.03 fold and 4R to 3R ASO decreased 4R-specific peptides 0.51 ± 0.04 fold compared with saline, *n* = 3 biological replicates/group.

These findings suggest that the presence of 4R tau in astrocytes leads them to take on a reactive, neurotoxic genetic profile consistent with their change in morphology. Still, it was unclear whether this phenotype was due to astrocytic uptake of tau protein or the expression of tau protein in astrocytes. Despite collective data in mice (24, 25) and humans (26–28) that support tau expression in astrocytes, the level of neuronal tau far exceeds astrocyte tau expression. To confirm that endogenous astrocytic tau protein is detectable, we performed mass spectrometry to analyze tau levels (Figure 2A). Tau protein was detectable in hTau mouse astrocytes by mass spectrometry, albeit at levels approximately 100-fold less than in neurons (Figure 2B). In addition, we detected total tau in primary hTau astrocytes via Western blot (Supplemental Figure 2C). We attributed these changes in tau isoforms to astrocytes alone, given that purity assessments of our astrocyte cultures did not suggest neuronal contamination (Supplemental Figure 3). These results are consistent with previous studies that have measured relative tau RNA expression levels (25, 28) and demonstrated the presence of endogenous tau in astrocytes.

### Manipulation of tau splicing toward more 4R tau promotes a neurotoxic astrocyte phenotype.

**Figure 3:**
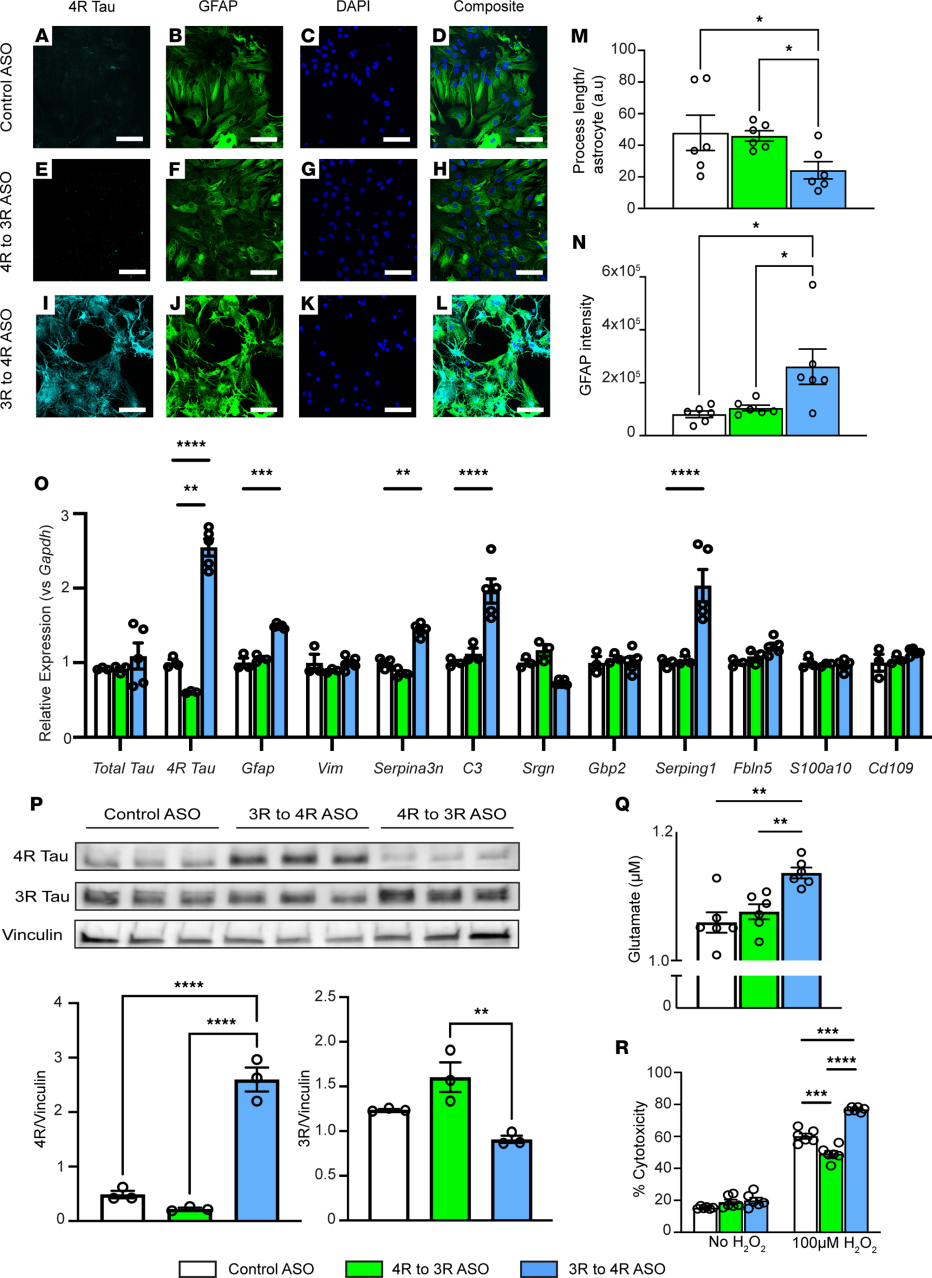
Increased 4R tau in primary hTau astrocytes induces a neurotoxic genetic signature and leads to dysfunction. (**A**, **E**, and **I**) 4R tau, (**B**, **F**, and **J**) GFAP, (**C**, **G**, and **K**) DAPI, and (**D**, **H**, and **L**) merged images from cultured hTau astrocytes treated with ASOs. Scale bar: 200 μm. (**M**) Average process length per astrocyte in hTau astrocytes treated with ASOs. Data are mean ± SEM, *P < 0.05 by 1-way ANOVA with multiple corrections, *n* = 6 biological replicates/treatment, 3 images per treatment. (**N**) GFAP intensity in hTau astrocytes treated with ASOs. Data are mean ± SEM, *P < 0.05 by 1-way ANOVA with multiple corrections, *n* = 6 biological replicates/treatment, 3 images per treatment. (**O**) Expression of select genes in cultured primary hTau astrocytes after ASO treatment measured by qRT-PCR. Data are normalized to *Gapdh* relative to control ASO levels and shown as mean ± SEM; 2-way ANOVA with Tukey’s multiple comparisons; *n* = 3–5 biological replicates/treatment, **P < 0.01, ***P < 0.001, ****P < 0.0001. (**P**) Western blots for 4R tau, 3R tau, and vinculin in hTau astrocytes treated with control ASO, 4R to 3R tau splicing, or 3R to 4R tau splicing ASO. Data are mean ± SEM; *n* = 3 biological replicates/treatment; 1-way ANOVA with Tukey’s multiple comparisons; **P < 0.01, ****P < 0.0001. (**Q**) Glutamate measured in cellular media after control, 3R to 4R tau splicing, or 4R to 3R tau splicing ASO treatment in hTau astrocytes. Data are mean ± SEM; *n* = 6 biological replicates/treatment; 1-way ANOVA with Tukey’s multiple comparisons; **P < 0.01. (**R**) Cytotoxicity (measured by LDH release) in control, 3R to 4R tau splicing, or 4R to 3R splicing ASO treated hTau astrocytes at baseline or following 100 μM H_2_O_2_ treatment. Data are mean ± SEM; *n* = 6 biological replicates/treatment; 2-way ANOVA with Tukey’s multiple comparisons; ***P < 0.001, ****P < 0.0001.

Despite observing a neurotoxic astrocytic signature in hTau mice following ASO-mediated tau splicing to increase 4R tau, the nonselective nature of ASO uptake prevented us from isolating cell type–specific changes. Therefore, we tested the effect of tau splicing manipulation in an in vitro astrocyte model. Primary hTau-expressing astrocytes from hTau mice were treated with either a control ASO, a 4R to 3R tau splicing ASO, or a 3R to 4R tau splicing ASO to identify if altering the ratio of tau isoforms in astrocytes alone would lead to similar changes as seen in vivo. First, we showed that the 3R to 4R tau splicing ASO increased 4R tau isoforms and that the 4R to 3R tau splicing ASO decreased 4R tau isoforms at the protein level using mass spectrometry analyses targeting 4R-specific peptides (Figure 2C). Compared with control or 4R to 3R tau splicing ASO-treated astrocytes (Figure 3, A–H, and Supplemental Figure 4), hTau astrocytes treated with the 3R to 4R tau splicing ASO (Figure 3, I–L) exhibited a retracting of processes toward the soma (Figure 3M) and brighter GFAP reactivity, both of which are associated with reactive astrocytes. Because these morphological changes were not evident in controls, we attribute this astrocyte reactivity to an increase in 4R tau as opposed to ASO toxicity.

hTau astrocytes treated with the 3R to 4R tau splicing ASO exhibited a significant increase in genes associated with a pan-reactive and neurotoxic genetic profile and no change in genes related to a neuroprotective phenotype as compared with astrocytes treated with the control ASO and the 4R to 3R tau splicing ASO (Figure 3O). As expected, mTau^–/–^ and C57BL/6 astrocytes treated with either the control ASO or the 3R to 4R tau splicing ASO showed no significant changes in any of the genes profiled (Supplemental Figure 5, A and B). Using 3R tau– and 4R tau–specific antibodies, we also identified a robust increase in 4R tau protein levels in hTau astrocytes treated with the 3R to 4R tau splicing ASO and a decrease in 3R tau levels compared with those treated with either the control ASO or the 4R to 3R tau splicing ASO (Figure 3P). Together with our in vivo findings, these in vitro gene profiles suggest increased 4R tau in astrocytes may bias them toward a reactive or neurotoxic phenotype.

### 4R tau expression in astrocytes hinders homeostatic functions.

Given the changes in astrocytic gene signatures, we tested if astrocyte functionality was altered following an increase in the 4R/3R tau ratio. As astrocytes are responsible for taking up excess glutamate at the synapse to prevent excitotoxicity, we tested whether an increased amount of 4R tau in astrocytes could lead to a decrease in glutamate uptake ability. We found that hTau astrocytes treated with the 3R to 4R tau splicing ASO exhibited a significantly reduced ability to take up glutamate from the cellular media (Figure 3Q) compared with the hTau astrocytes treated with the control ASO or the 4R to 3R tau splicing ASO. mTau^–/–^ astrocytes treated with either the control or the 3R to 4R tau splicing ASO exhibited no change in glutamate uptake ability (Supplemental Figure 6). These data suggest that the increased levels of 4R tau led to a decrease in astrocytic ability to recycle excitatory neurotransmitters.

In many neurodegenerative diseases, the loss of astrocytic ability to protect neurons from oxidative damage leads to disease progression (29–31). Therefore, we examined whether altering the ratio of tau isoforms affected the vulnerability of astrocytes to oxidative stress. Following exposure to hydrogen peroxide, a higher level of LDH release was detected in both hTau and mTau^–/–^ astrocytes regardless of treatment, indicative of cytotoxicity; however, hTau astrocytes treated with the 3R to 4R tau splicing ASO exhibited significantly higher LDH release levels than hTau astrocytes treated with the control ASO and the 4R to 3R tau splicing ASO (Figure 3R). We also identified that primary neurons cocultured with 4R tau–expressing hTau astrocytes were more prone to death after hydrogen peroxide treatment (Supplemental Figure 7). Taken together, these data suggest that increased 4R tau levels in astrocytes are sufficient to cause functional deficiencies.

### Human astrocytes expressing 4R tau exhibit a neurotoxic phenotype and a disruption to homeostatic function.

**Figure 4:**
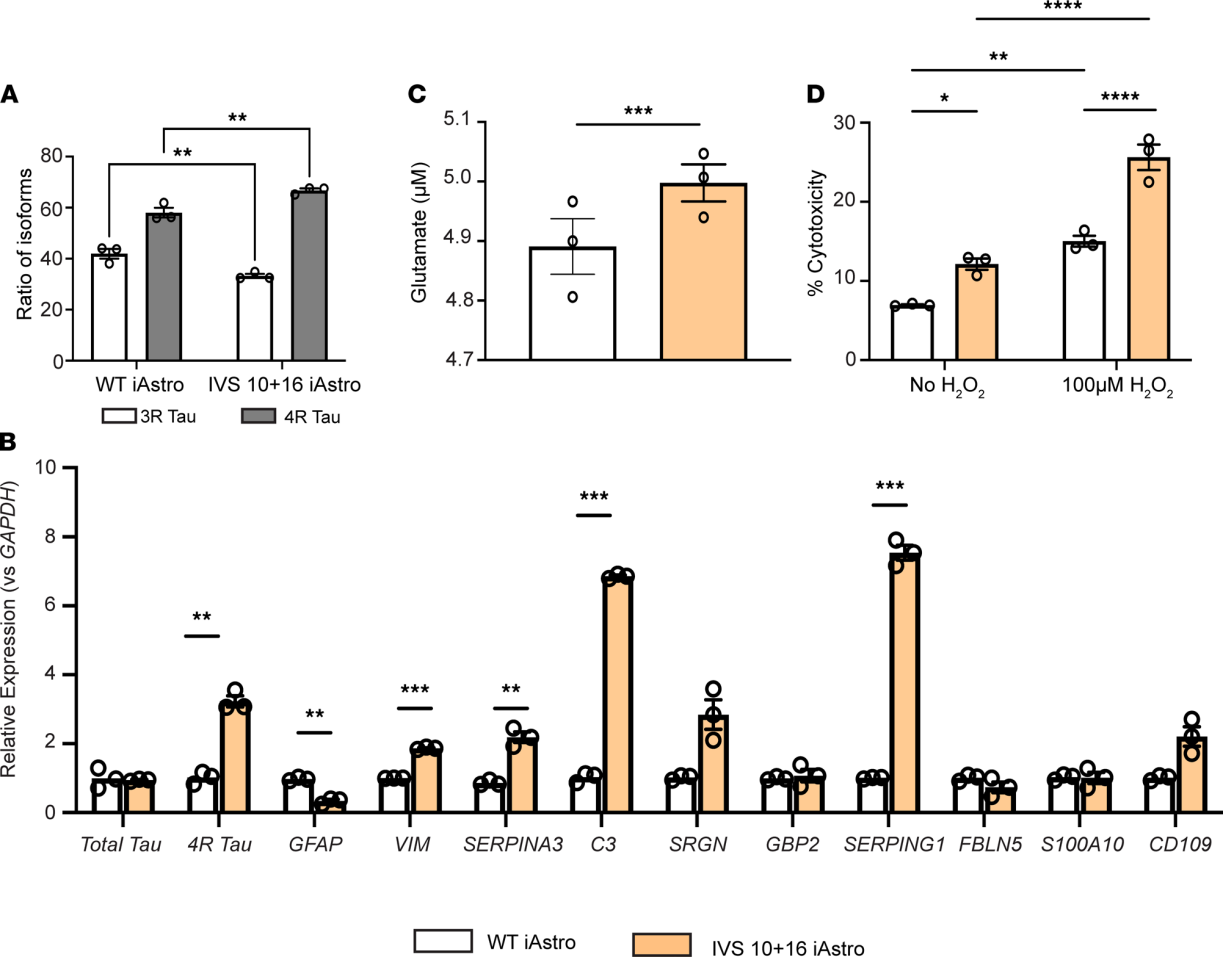
iAstrocytes exhibit a neurotoxic phenotype and disruption to homeostatic function. (**A**) Relative levels of 3R and 4R mRNA tau in iAstrocytes. Data are mean ± SEM; *n* = 3 biological replicates/treatment. (**B**) Expression of select pan-reactive (*GFAP*, *Vimentin*, and *SERPINA3*), neurotoxic (*C3*, *SRGN*, *GBP2*, *SERPING1*, and *FBLN5*), and neuroprotective (*S100a10* and *CD109*) genes in WT and IVS 10+16 *MAPT* mutation iAstrocytes measured by qRT-PCR. Data are normalized to *GAPDH* relative to isogenic levels and shown as mean ± SEM; 1-way ANOVA with Tukey’s multiple comparisons; *n* = 3 wells; **P < 0.01, ***P < 0.001. (**C**) Glutamate concentration measured in cellular media in iAstrocytes. Data are mean ± SEM; *n* = 3 biological replicates/group; unpaired, 2-tailed *t* test; ***P < 0.001. (**D**) Cytotoxicity (measured by LDH release) in iAstrocyte cultures at baseline and following 100 μM H_2_O_2_ treatment. Data are mean ± SEM; *n* = 3 biological replicates/group; 2-way ANOVA with Tukey’s multiple comparisons; *P < 0.05, **P < 0.01, ****P < 0.0001.

While the results above using a 3R to 4R splicing ASO strongly suggest that the astrocyte toxicity was caused by increased 4R tau, we cannot entirely exclude an off-target, ASO-related effect. Therefore, we used induced pluripotent stem cell–derived (iPSC-derived) astrocytes (iAstrocytes) from fibroblasts collected from patients with an IVS 10+16 *MAPT* mutation to investigate 4R tau–mediated effects independent of ASO interventions. These cells express 4R tau immediately following differentiation, making them an excellent model to study how increased 4R tau alone affects cellular function (32). We showed increased 4R tau expression in IVS 10+16 iAstrocytes, which was not evident in the corrected isogenic iAstrocyte controls (Figure 4, A and B). In addition, we identified that, at baseline, IVS 10+16 iAstrocytes expressed higher levels of pan-reactive and neurotoxic genes than isogenic control iAstrocytes (Figure 4B). Upregulated genes were similar to those identified in both hTau mice and primary hTau-expressing astrocytes treated with the 3R to 4R tau splicing ASO, providing further support for increased 4R tau leading to a neurotoxic astrocytic phenotype. When tested for functional responses, iAstrocytes derived from IVS 10+16 mutation carriers were significantly less able to take up glutamate from the media than control iAstrocytes (Figure 4C). When we examined the iAstrocytes’ ability to respond to oxidative stress, we found that IVS 10+16 iAstrocytes were more vulnerable at baseline, which was exacerbated upon hydrogen peroxide stress (Figure 4D). These results provide a human correlate for the neurotoxicity and loss of homeostatic function in astrocytes with increased 4R tau and suggest that tau in astrocytes may be important for neurodegenerative disease progression.

### Human astrocytes expressing 4R tau increase neuronal excitability.

**Figure 5:**
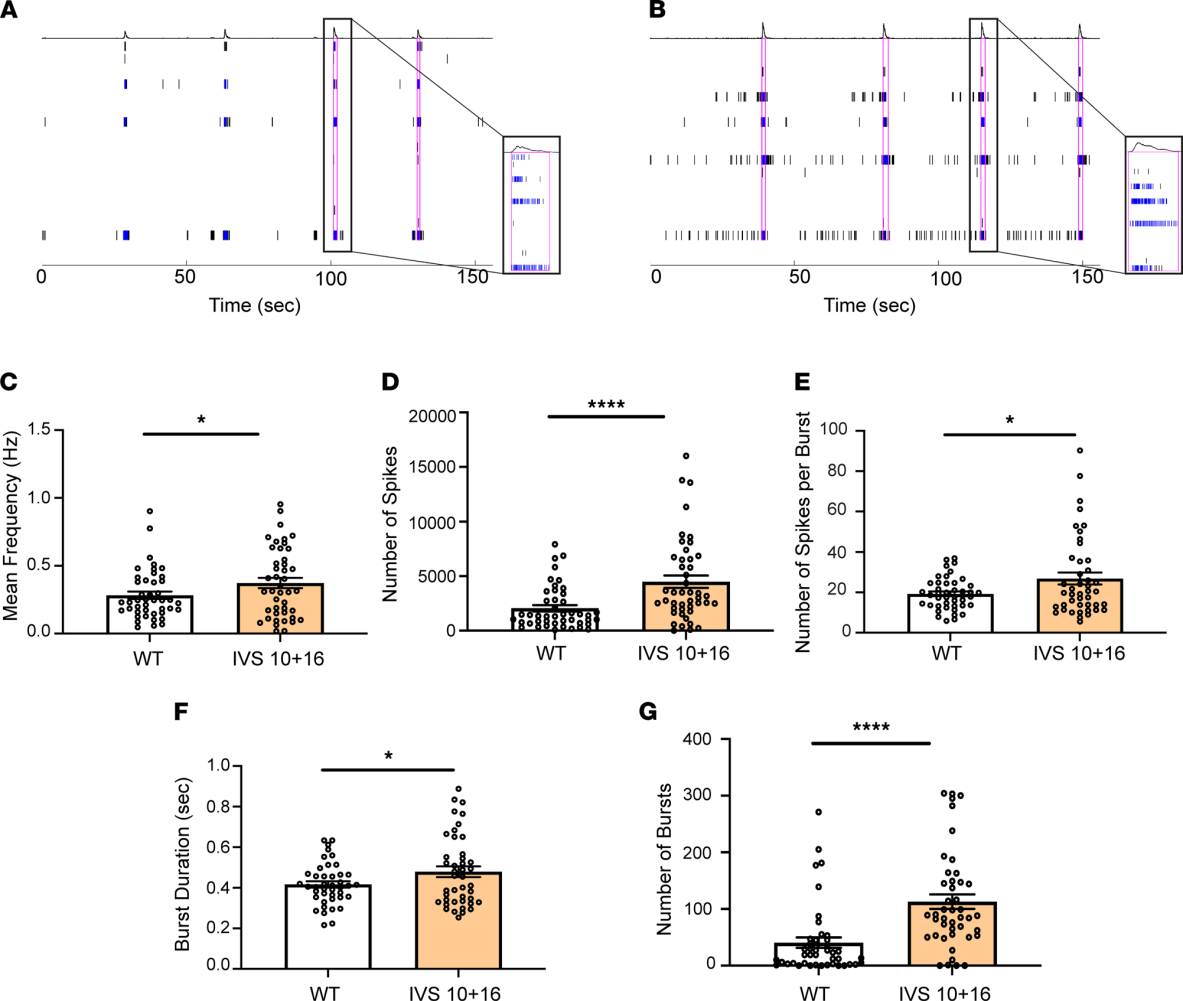
Homeostatic control of neuronal excitability is reduced in 4R tau–expressing iAstrocytes. Representative raster plots of burst rates from neurons cocultured with (**A**) WT or (**B**) IVS 10+16 *MAPT* mutation iAstrocytes. The blue tick marks represent spikes that were part of a single electrode firing, while black tick marks represent multi-electrode firings. The magenta outlines indicate network bursts. (**C**) Mean frequency, (**D**) number of spikes, (**E**) number of spikes per burst, (**F**) burst duration, and (**G**) number of bursts were measured from neurons cocultured with WT or IVS 10+16 iAstrocytes. Data are mean ± SEM; *n* = 45 wells/group and 3 recordings; *P < 0.05, ****P < 0.0001 by unpaired, 2-tailed *t* test.

We observed that 4R tau–expressing astrocytes could not take up glutamate from the media, which we hypothesized may exacerbate neuronal hyperexcitability. To test this, we cultured mixed cortical iPSC-derived neurons with IVS 10+16 mutation carrier iAstrocytes or isogenic control iAstrocytes and measured spontaneous neuronal activity (Figure 5, A and B). Neurons cocultured with IVS 10+16 iAstrocytes exhibited a significantly higher firing rate (Figure 5C), more signals being sent (Figure 5D), a higher number of signals per burst (Figure 5E), more extended burst duration (Figure 5F), and more bursts (Figure 5G) than those cultured with isogenic control iAstrocytes. The higher number of bursts in neurons cocultured with 4R tau–expressing iAstrocytes implies that the neurons were firing in hypersynchrony, while the increase in spike number and firing rate suggests that the neurons were firing more frequently when cultured with 4R tau–expressing iAstrocytes. These data indicate that when astrocytes express higher levels of 4R tau, neurons near those astrocytes demonstrate hyperexcitability and may potentiate neurotoxicity.

### Lowering levels of total tau in 4R tau–expressing iAstrocytes rescues human astrocyte function.

**Figure 6:**
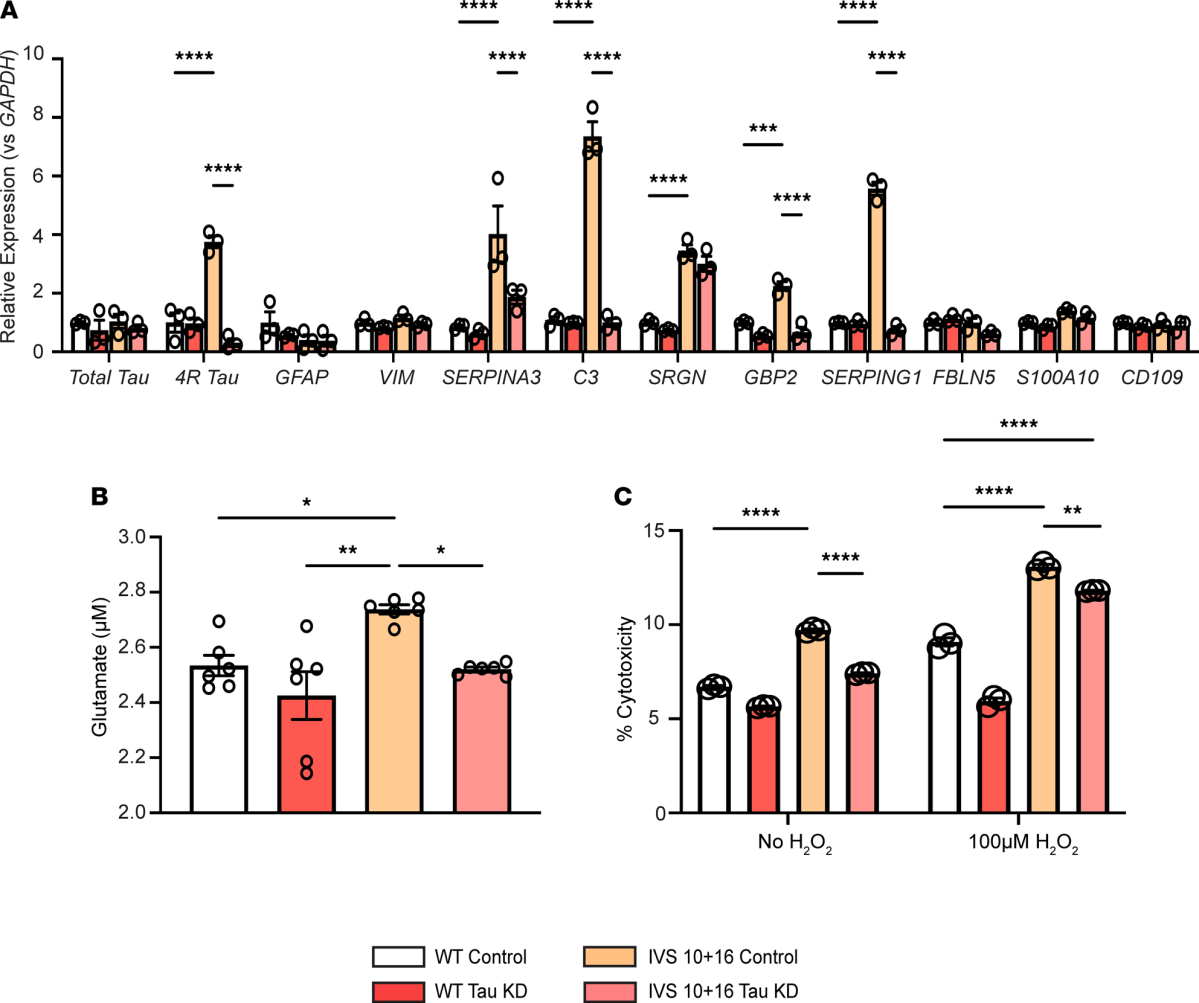
Lowering levels of total tau in iAstrocytes rescues neurotoxic phenotype and function. (**A**) Expression of select pan-reactive (*GFAP*, *Vimentin*, and *SERPINA3*), neurotoxic (*C3*, *SRGN*, *GBP2*, *SERPING1*, and *FBLN5*), and neuroprotective (*S100a10* and *CD109*) genes in WT or IVS 10+16 *MAPT* mutation iAstrocytes treated with control ASO or tau-knockdown (tau-KD) ASO measured by qRT-PCR. Data are normalized to *GAPDH* relative to WT levels and shown as mean ± SEM; *n* = 3 biological replicates/treatment; 2-way ANOVA with Tukey’s multiple comparisons; ***P < 0.001, ****P < 0.0001. (**B**) Glutamate concentration measured in cellular media in iAstrocytes treated with control ASO or tau-KD ASO. Data are mean ± SEM; *n* = 6 biological replicates/treatment; 1-way ANOVA; *P < 0.05, **P < 0.01. (**C**) Cytotoxicity (measured by LDH release) in iAstrocyte cultures treated with a control ASO or tau-KD ASO at baseline and following 100 μM H_2_O_2_ treatment. Data are mean ± SEM; *n* = 3 biological replicates/treatment; 2-way ANOVA with Tukey’s multiple comparisons; **P < 0.01, ***P < 0.001.

Having identified that increased 4R tau expression is detrimental to homeostatic human astrocyte function, we hypothesized that lowering the amount of total tau expressed in iAstrocytes alone would also improve function. While specifically reducing 4R tau in these astrocytes would be ideal, the previously developed version of the 4R to 3R tau splicing ASO that was initially used in the study was not efficient in IVS 10+16 iAstrocytes (Supplemental Figure 8), likely due to the additional secondary structure created by the mutation that prevented ASO binding. Therefore, we used a total tau mRNA–lowering approach (33, 34), which is effective at reversing tau pathology and reducing seizure severity (34). We treated IVS 10+16 mutation iAstrocytes with a tau mRNA–lowering ASO and measured mRNA expression, glutamate uptake ability, and response to oxidative stress. We found that following a reduction of total tau levels, select pan-reactive and neurotoxic genes were significantly downregulated (Figure 6A), suggesting that the reactive phenotype was rescued with tau mRNA lowering. We also identified that IVS 10+16 iAstrocytes with lowered total tau were better able to take up glutamate from the media (Figure 6B) compared with those treated with the control ASO. Lowering total tau levels also allowed the iAstrocytes to resist hydrogen peroxide–induced oxidative stress, as exhibited by the decrease in cytotoxicity (Figure 6C). These data strongly suggest that by lowering tau levels in astrocytes alone, we can prevent astrocyte reactivity and rescue astrocytes’ ability to perform homeostatic functions.

### Lowering levels of total tau in 4R tau–expressing iAstrocytes rescues neuronal excitability.

**Figure 7:**
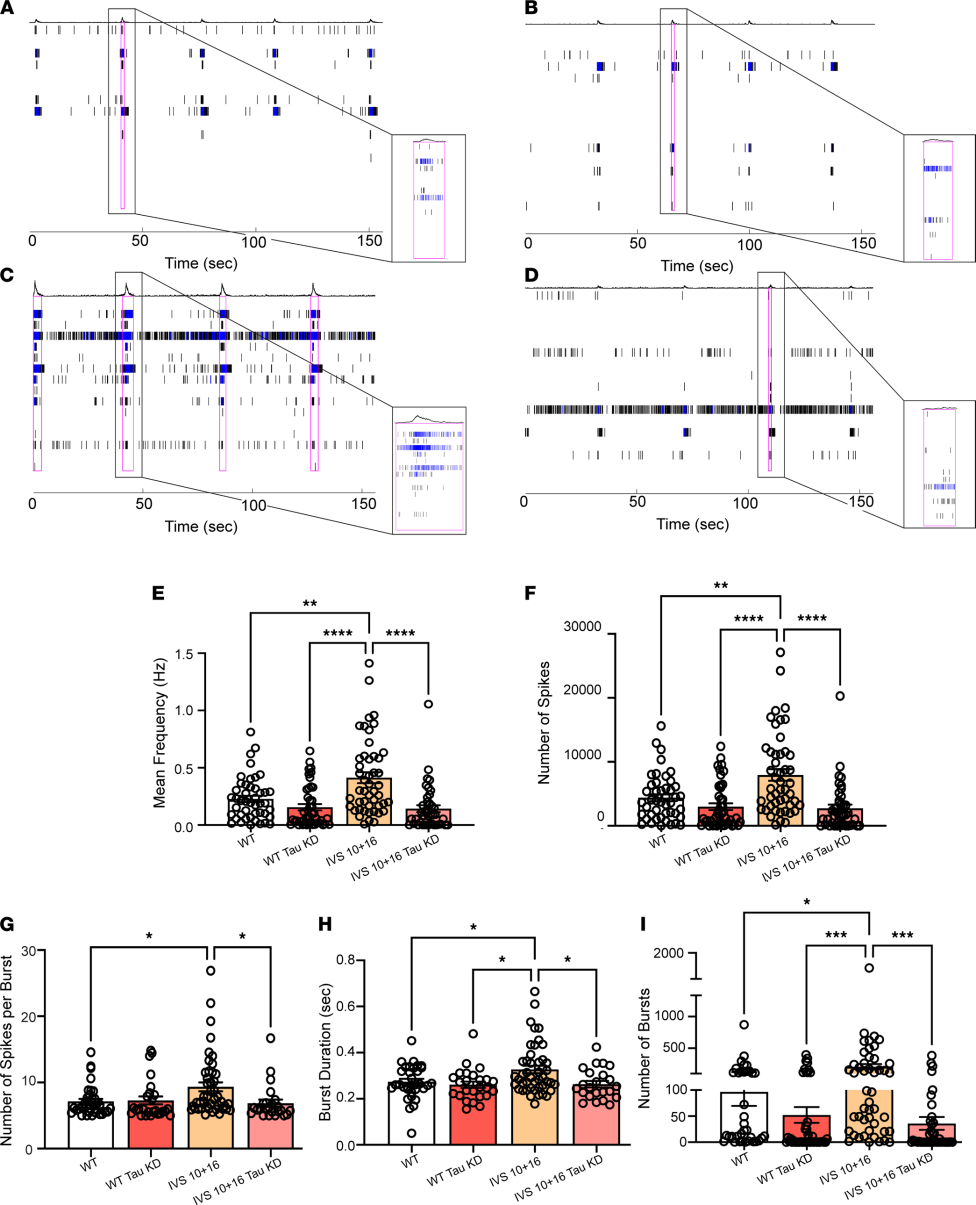
Homeostatic control of neuronal excitability is rescued following lowering of tau levels in 4R tau–expressing iAstrocytes. Representative raster plots of burst rates from neurons cocultured with (**A**) WT, (**B**) WT tau-KD ASO-treated, (**C**) IVS 10+16 *MAPT*, and (**D**) IVS 10+16 *MAPT* tau-KD ASO-treated iAstrocytes. The blue tick marks represent spikes that were part of a single electrode firing, while black tick marks represent multi-electrode firings. The magenta outlines indicate network bursts. (**E**) Mean frequency, (**F**) number of spikes, (**G**) number of spikes per burst, (**H**) burst duration, and (**I**) number of bursts were measured from neurons cocultured with WT, WT tau-KD ASO-treated, IVS 10+16 *MAPT*, or IVS 10+16 *MAPT* tau-KD ASO-treated iAstrocytes. Data are mean ± SEM; *n* = 30–45 wells/group and 3 recordings; *P < 0.05, **P < 0.01, ***P < 0.001, ****P < 0.0001, by 1-way ANOVA with multiple corrections.

We then investigated if lowering total tau would prevent astrocytic 4R tau–induced neuronal hyperexcitability. We pretreated the iAstrocytes with either control or total tau-reducing ASO before coculturing with iPSC-derived neurons to measure spontaneous neuronal activity (Figure 7, A–D). We found that neurons cocultured with the IVS 10+16 iAstrocytes treated with total tau-lowering ASO prevented increases in neuronal firing rate, number of signals being sent, number of bursts, signals per burst, and burst duration compared with control ASO-treated IVS 10+16 iAstrocytes (Figure 7, E–I). We detected no change in neuronal firing when the isogenic iAstrocytes were treated with the total tau-lowering ASO as compared to the isogenic iAstrocytes treated with the control ASO (Figure 7, E–I). These results suggest that the presence of 4R tau in astrocytes is sufficient to induce neuronal hyperexcitability, which can be rescued by tau lowering.

### Lowering levels of total tau in 4R tau–expressing iAstrocytes improves neuronal survival.

**Figure 8:**
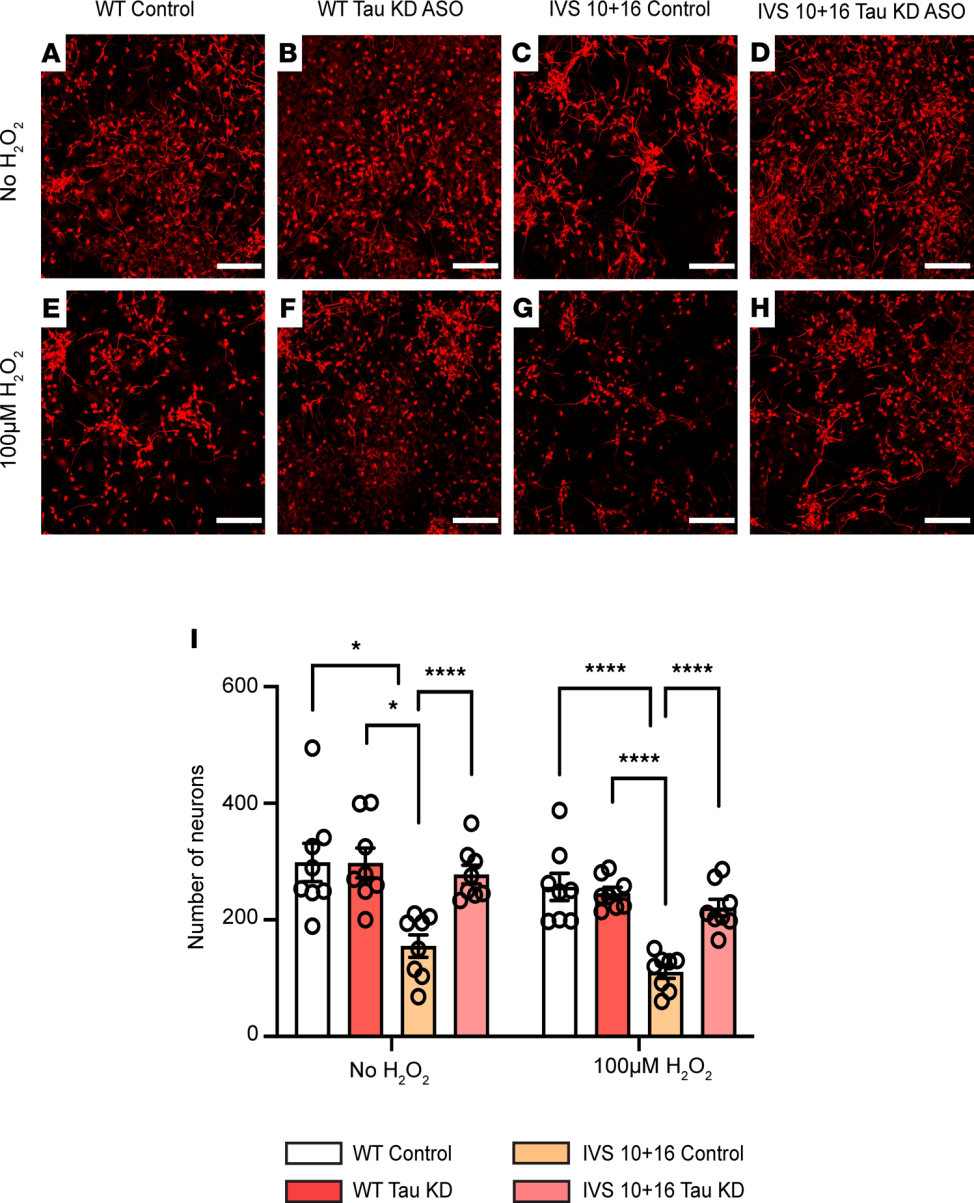
Increased 4R tau expression in iAstrocytes leads to neuronal death. (**A**–**H**) Representative images of microtubule associated protein 2 staining in iPSC cortical neurons cocultured with WT or IVS 10+16 iAstrocytes treated with either control or tau-KD ASO at baseline and after hydrogen peroxide treatment. Scale bar: 200 μm. (**I**) Quantification of the number of neurons in cocultures. Data are shown as mean ± SEM; *n* = 8 biological replicates per treatment and 10 images per replicate: *P < 0.05, ****P < 0.0001 by 2-way ANOVA with multiple corrections.

Given that we identified a marked increase in neuronal hyperexcitability when neurons were cocultured with 4R tau–expressing astrocytes, we wanted to test whether this affected neuronal survival. We again treated iAstrocytes derived from an IVS 10+16 mutation carrier or their isogenic control with a control or tau-lowering ASO prior to coculturing them with iPSC-derived cortical neurons. We found no reduction in the number of neurons when cocultured with the isogenic control iAstrocytes treated with the control ASO or the tau-lowering ASO at baseline (Figure 8, A, B, and I). In contrast, we saw a significant decrease in neuronal numbers when cocultured with the IVS 10+16 iAstrocytes treated with the control ASO (Figure 8, C and I). This decrease was reversed when neurons were cocultured with the IVS 10+16 iAstrocytes treated with the total tau-lowering ASO (Figure 8, D and I), suggesting that reducing tau in the 4R tau–expressing astrocytes can increase neuronal survival. As we had previously seen that astrocytes that expressed higher tau levels were more vulnerable to oxidative stress, we tested if this vulnerability would lead to an increase in neuronal death. As expected, we observed a decrease in the number of neurons’ cultures treated with hydrogen peroxide to induce stress (Figure 8, E–I). The cocultured neurons with the IVS 10+16 iAstrocytes treated with the control ASO had the greatest loss of neuronal number (Figure 8, G and I). This decrease was reversed when the iAstrocytes were pretreated with the tau-lowering ASO (Figure 8, H and I). These results demonstrate that 4R tau–expressing astrocytes are less neuroprotective at baseline and under stress, but lowering the levels of tau in these astrocytes is sufficient to reduce neuronal death.

## Discussion

The source and pathogenic mechanisms of tau in neurodegenerative diseases are primarily attributed to neurons despite concurrent tau accumulation in glial cells. Here, we demonstrate that even low levels of tau, specifically 4R tau, in astrocytes are sufficient to disrupt normal astrocytic function and may contribute to neuronal dysfunction in disease. Primary astrocytes with increased 4R tau exhibited reactive gene expression, reduced ability to take up glutamate, and increased sensitivity to oxidative stress, which were all mitigated by tau mRNA reduction. Human astrocytes derived from IVS 10+16 *MAPT* mutation carriers, which independently express elevated 4R tau, showed a similar dysfunctional phenotype that reversed with tau reduction. Further, neurons cultured with IVS 10+16 *MAPT* astrocytes exhibited hyperexcitability and increased levels of death, suggesting 4R tau in astrocytes may promote aberrant neuronal responses, which could also be mitigated by tau reduction. The correlations from in vivo hTau mice to isolated astrocytes and iPSC-derived astrocytes are striking and strongly suggest that increased 4R tau affects astrocytes. Further, the results from iPSC-derived astrocytes show that the effects are independent of ASO treatment. Together, these data support the conclusion that increased expression of 4R tau by astrocytes causes a reactive astrocytic phenotype and is detrimental to their ability to perform homeostatic functions.

Tau pathology is most commonly associated with neurons, but astrocytes also exhibit pathologic tau deposition in many primary tauopathies (13), even in the absence of detectable neuronal tau pathology (35–37). Tau deposition within astrocytes often serves as a defining feature for several primary tauopathies, including progressive supranuclear palsy, corticobasal degeneration, and argyrophilic grain disease, aiding in their diagnoses (13, 38). Selective deposition of tau isoforms within neurons traditionally confers the 3R or 4R tauopathy classification. However, astrocytes tend to exhibit higher 4R tau deposition, even in 3R tauopathies such as Pick’s disease (39, 40), suggesting that a component of astrocytic biology may predispose them to express or deposit more 4R tau. Aging-related tau astrogliopathy (ARTAG) has been used to classify diseases where tau deposition occurs in astrocytes throughout the brain (26), and many primary tauopathies exhibit ARTAG-related astrocyte morphologies (12).

While there is tau deposition in astrocytes in primary tauopathies, the source of astrocytic tau — whether endogenous to astrocytes or taken up from neighboring neurons or oligodendrocytes (37) — is unclear. We show that in astrocytes isolated from hTau mice and derived from human iPSCs, altered endogenous tau isoform levels promote reactive, dysfunctional astrocytes, which may predispose tau to deposit (41–43). Prior studies in our lab have shown that an ASO-mediated increase in 4R tau in hTau mice leads to tau phosphorylation and aggregation (17). However, we did not attribute the tau pathology to neurons versus astrocytes. Our in vitro studies here have allowed us to investigate the role of tau expression in astrocytes separate from neurons; however, we cannot exclude a neuronal contribution for pathological tau in astrocytes, as it has been well documented that astrocytes may take up various forms of extracellular tau and contribute to tau spread (36, 37, 44–46). Once tau is internalized, it disrupts normal astrocytic functions, including calcium signaling and gliotransmitter release (47), and impairs glutamate clearance (48, 49). Future studies will be needed to probe cell type–specific and overlapping mechanisms of tau pathology in neurodegenerative disease.

Previously published studies have relied on tau overexpression to assess tau isoforms in astrocytes (24, 50). 4R tau–expressing astrocytes isolated from P301S mice were less able to support neurons in vitro (51). Additionally, in a fruit fly model of tauopathy, 4R tau expression led to greater neurodegeneration and impairment in learning in memory (8). In a separate study, 3R tau overexpression in astrocytes was sufficient to cause memory deficits and neuronal dysfunction in mice (24). These studies are challenging to interpret in the context of human disease as overexpression of normal tau is linked to the accumulation of filamentous actin, formation of tau tangles, and neurodegeneration (1, 42, 52–54). Here, we chose to alter the ratios of endogenously expressed physiological levels of astrocytic tau. The tau splicing ASOs used do not alter total tau levels but instead alter the ratio of 3R tau to 4R tau (17) by interfering with the alternative splicing of exon 10. Our data suggest that 4R tau expression in astrocytes promotes a reactive astrocytic genetic and morphological phenotype and leads to the loss of their ability to protect neurons, making tau in astrocytes a potential contributor to tau-related neurodegenerative disease mechanisms. Further, we identified that human 4R tau–expressing astrocytes displayed a loss in glutamate uptake ability and, when cultured with cortical neurons, induced neuronal hyperexcitability and hypersynchrony. These in vitro findings may explain the elevated seizure phenotype identified in vivo with increased 4R tau (17). Our data suggest that the presence of 4R tau in astrocytes leads to astrocyte dysfunction consistent with neurodegenerative diseases.

While astrocytes’ role in neurodegenerative disease progression is still not well understood, astrocytes are responsible for maintaining neuronal health and homeostasis under physiological settings (55). Therefore, when astrocyte function is disrupted, as occurs in Alzheimer’s disease, neurons may be less protected from toxic proteins, stress, or inflammation. The loss of glutamate buffering ability in reactive astrocytes may lead to Alzheimer’s disease progression (18, 31, 56, 57). In disease, astrocytes may also begin to secrete proinflammatory cytokines that contribute to disease progression and may exhibit upregulation of neurotoxic genes (23). Astrocytes in disease also exhibit increased sensitivity to oxidative damage (29) and impaired glutamate buffering ability at the synapse (18, 57). Therefore, dysfunctional astrocytes, possibly resulting from tau accumulation, may contribute to disease progression.

An upregulation of *Serpina3n*, *C3*, and *Serping1* was seen in each of our models; it is possible that we have identified a new astrocytic phenotype that can be associated with increased 4R tau expression and neuronal hyperexcitability. These genes have been associated with synaptic dysfunction, and it has been suggested that increased levels of *Serpina3n* can lead to increased expression of *C3* and *Serping1* (58, 59). It has also been shown that seizures, neuronal loss, and memory deficits can be reduced by lowering the expression levels of *Serpina3n* (59). This finding suggests that there may be a link between 4R tau expression and the role of astrocytes in aspects of neurodegenerative disease.

Collective data, including from our lab, support the therapeutic benefit of tau-lowering strategies in tauopathy. Given that many primary tauopathies have mutations in and around exon 10 (11, 14), which encodes 4R tau, tau splicing strategies that lower 4R tau may also be viable therapeutically. We investigated a *MAPT* mutation (IVS 10+16), which causes an increased expression of 4R tau in humans (60). Due to the location of the mutation at the binding site of the 4R to 3R tau splicing ASO, we were unable to manipulate 4R tau levels specifically. However, lowering total tau levels using a total tau knockdown ASO was able to reduce expression of reactive astrocytic genes, increase astrocytic ability to take up excess glutamate, and prevent cell death after oxidative stress. Tau expression in neurons is a clear target for tau-lowering strategies, but these new data support astrocyte tau in disease progression, despite its low level of expression. We show, for the first time to our knowledge, that 4R tau–mediated dysfunction in astrocytes can be reversed by lowering the levels of either 4R tau or total tau in astrocytes. Additionally, when tau was reduced in astrocytes alone, it led to a decrease in neuronal hyperexcitability, suggesting that cell-specific tau lowering in astrocytes may be a viable therapeutic strategy. Together, these data strongly support that tau-lowering ASOs that are currently in a clinical trial for Alzheimer’s disease may be able to rescue neuronal and astrocyte functions in neurodegenerative disease.

## Methods

### Mass spectrometry.

Immunoprecipitation and mass spectrometry were performed as previously described with some modifications (61, 62). Briefly, cultured astrocytes and neurons were collected in phosphate-buffered saline (PBS), pelleted, and frozen until analyses. Cell pellets were lysed with detergent and caotropic reagents (final 1% NP-40, 5 mM guanidine) and subjected to immunoprecipitation with Tau1, HJ8.5, and HJ8.7 tau antibodies. The immunoprecipitated tau was digested by trypsin, desalted with Toptip C18 column (Glygen Corporation), and analyzed by the high-resolution mass spectrometer Orbitrap Eclipse (Thermo Fisher Scientific). Internal standard, ^15^N-uniform labeled 2N4R recombinant tau was spiked into the samples for normalization. The mid domain peptide 212-221 was used to represent the relative amount of tau in astrocytes and neurons. 4R isoform–specific peptides 282-290, 275-280, and 299-317 and 3R 4R common peptide 260-267 were measured to assess the effects of 3R to 4R and 4R to 3R ASO treatments.

### Animals.

Age- and sex-matched male and female hTau-expressing (*hTau*) (available at Jackson Laboratory, stock number 005491) (16), mTau-knockout (*mTau^–/–^*) (available at Jackson Laboratory, stock number 004779) (17, 63), and *C57BL/6* mice (available at Jackson Laboratory, stock number 000664) were aged to 12 months prior to experimental treatment.

### ASOs.

All ASOs were designed with a phosphorothioate backbone and either uniform or “gapmer” 2′-*O*-methoxyethyl sugar ring modifications to achieve tau splicing or knockdown, respectively (34, 64). The fully modified design prevents target degradation, while the gapmer design, consisting of 10 central, unmodified nucleotides flanked by 5 modified nucleotides, enables RNaseH-mediated target degradation. An ASO control similar in structure and modifications but without target specificity was included in experimental treatments to account for potential toxicity or off-target effects. All ASOs were synthesized by Ionis Pharmaceuticals and provided for use. Sequences are as follows: tau splicing control ASO (5′-TCATTTGCTTCTACAGG-3′), 3R to 4R tau splicing ASO (5′-GGCGCATGGGACGTGTGA-3′), 4R to 3R tau splicing ASO (5′-GGACGTGTGAAGGTACTC-3′), tau-knockdown control ASO (5′-GCTTTTACTGACCATGCGAG-3′), and tau-knockdown ASO (5′-CCTTCCCTGAAGGTTCCTCC-3′). For in vitro experiments, ASOs were diluted to 10 μM in astrocyte media and replaced every other day for 12 days. hTau astrocytes treated with a control ASO exhibited similar levels of select mRNA gene expression compared to those that were treated with saline-supplemented media (data not shown); therefore, a control ASO was used for all subsequent treatments. For in vivo experiments, ASOs were continuously administered to the right lateral ventricle via osmotic pump (ALZET) as described below.

### Intraventricular delivery of ASOs.

Osmotic pumps were prepared as previously described (17, 34). Mice were anesthetized using a constant flow of 2%–3% inhalant isoflurane and placed onto a stereotaxic head frame (Kopf). While anesthetized, the scalp was cut, and a pump was placed into a subcutaneous pocket above the scapula. A metal catheter attached to the pump via flexible tubing was positioned to 1.1 mm lateral, 0.5 mm posterior, and 2.5 mm ventral from bregma and secured to the skull to prevent movement. The incision was then sutured, and mice were placed on a 37°C warming pad until ambulatory. The dose of ASO used for splicing studies was 15 μg/d for 3R to 4R tau splicing and control ASOs. Following the surgical procedure, all mice were individually housed for 2 months until euthanasia.

### Primary astrocyte cultures.

Cortices from hTau, mTau^–/–^ littermate, and C57BL/6 mouse pups (P2–P4) were isolated as previously described (65) and cultured until confluence on 100 μg/mL poly-d-lysine–coated (Corning CB-40210) plates in primary astrocyte media (DMEM, 10% heat-inactivated FBS, 1× penicillin-streptomycin) at 37°C, 5% CO_2_. The mixed glial cultures were purified by shaking at 270 rpm for 6 hours at 37°C to achieve a pure primary astrocyte culture (Supplemental Figure 8).

### Primary neuron isolation.

Cortices from E15.5 C57BL/6 mouse pups were isolated as previously described (66). Briefly, the cortices were isolated and the meninges removed; the cortices were then digested in 0.05% trypsin for 15 minutes. A single-cell suspension was made, and the cells were counted. For coculture experiments, 150,000 neurons/mL were plated on top of hTau astrocytes. The following day, 1 μM of Ara-C was added to prevent glial contamination.

### Primary neuron and astrocyte cocultures.

Cortices from hTau, mTau^–/–^ littermate, and C57BL/6 mouse pups (P2–P4) were isolated as described above and cultured until confluence on 100 μg/mL poly-d-lysine–coated (PDL-coated) (Corning CB-40210) plates in primary astrocyte media (DMEM, 10% heat-inactivated FBS, 1× penicillin-streptomycin) at 37°C, 5% CO_2_. The mixed glial cultures were purified by shaking at 270 rpm for 6 hours at 37°C to achieve a pure primary astrocyte culture. The purified astrocytes were treated with ASOs for 12 days prior to coculture with primary neurons. These cells were cultured in primary neuronal media (Neurobasal, B27, N2, 1× glutamax, and 1× penicillin-streptomycin). The cocultures were grown for 14 days to allow for the formation of mature synapses. On day 14, the media were supplemented with saline or 100 μM H_2_O_2_ for 24 hours prior to fixation and immunocytochemical analysis.

### iAstrocyte differentiation and culture.

iPSCs (GIH-36 C2 [IVS 10+16/WT] and GIH-36 C2 IVS10+16 1D01 [WT/WT]) (67) were provided by Washington University in St. Louis for use. The cells were cultured in neural induction media for 2 weeks to promote a neural lineage. The neural progenitor cells were then cultured for 30 days in astrocyte media as per established protocol (68) in the Human Cells, Tissues and Organoid Core at Washington University in St. Louis. iAstrocytes were used for experiments after 60 days in culture.

### iPSC-derived neuron and astrocyte cocultures.

iAstrocytes were cultured as described above and treated with ASOs for 12 days prior to coculture with iPSC-derived mixed cortical neurons (BrainXell catalog BX-0500). The neurons were plated at a 5:1 ratio to iAstrocytes in seeding media supplemented with the astrocyte supplement (BrainXell catalog BX-2600). The following day, a half media change was done using the day 1 medium. On day 4, an additional half change of media was done, and this medium was supplemented with day 4 supplement (BrainXell). From day 7 onward, a half media change was done every 3–4 days. Media composition is listed in Supplemental Table 3. After 12 days of culture, the cells were treated with media supplemented with either saline or 100 μM H_2_O_2_ for 24 hours prior to fixation and immunocytochemical analysis.

### Immunoblotting.

Cells were lysed in cold RIPA lysis buffer supplemented with complete EDTA-free protease inhibitor cocktail (Roche Diagnostics). Protein lysate concentrations were measured using the Pierce BCA Protein Assay Kit (Thermo Fisher Scientific), and 20 μg of protein was separated using a 4%–20% polyacrylamide gel (Bio-Rad) and transferred onto a PVDF membrane. The membrane was probed for tau 5 1:1000 (Abcam catalog ab3931), GAPDH 1:1000 (Cell Signaling Technology catalog 2118), 3R 1:1000 (MilliporeSigma catalog 05-803); 4R 1:1000 (Cosmo Bio catalog CAC-TIP-4RT-P01), or Vinculin 1:1000 (MilliporeSigma catalog V9131), overnight, then incubated with secondary HRP antibodies (all at 1:5000; ECL anti–mouse IgG catalog NA931V or anti–rabbit IgG catalog NA934V, GE Healthcare, now Cytiva). The membrane was imaged using a SYNGENE reader.

### Immunofluorescence staining.

Free-floating 50 μm thick tissue sections were washed in 1× Tris-buffered saline before blocking in 5% normal horse serum diluted in 0.1% Triton X-100. The sections were incubated in primary antibody (3R 1:1000, MilliporeSigma catalog 05-803; 4R 1:1000, Cosmo Bio catalog CAC-TIP-4RT-P01; GFAP 1:1000, MilliporeSigma catalog AB5541) at 4°C overnight. The next day the sections were incubated in the blocking buffer prior to incubation with fluorescently tagged secondary antibodies (anti-mouse Alexa Fluor 594, Thermo Fisher Scientific catalog A-21125; anti-rabbit Alexa Fluor 647, Thermo Fisher Scientific catalog A32795; or anti-chicken Alexa Fluor 488, Jackson ImmunoResearch catalog 703-545-155; all at 1:500). Sections were then mounted and sealed with Fluoromount media (Southern Biotech). The Nikon A1Rsi confocal microscope was used for acquiring images. Colocalization of 4R tau and GFAP was analyzed using ImageJ (NIH).

### Immunocytochemical staining.

Cells were fixed using 4% paraformaldehyde (Santa Cruz Biotechnology catalog sc-281692) and permeabilized using 0.1% Triton X-100 in PBS. The cells were then incubated in blocking buffer (0.5% BSA diluted in PBS). The cells were incubated in primary antibody (3R 1:1000, MilliporeSigma catalog 05-803; 4R 1:1000, Cosmo Bio catalog CAC-TIP-4RT-P01; GFAP 1:1000, MilliporeSigma catalog AB5541, MAP2 1:1000 Abcam catalog 5392, S100β 1:100 Abcam catalog ab52642, IBA1 1:1000, FUJIFILM Wako Pure Chemical Corporation catalog 019-19741, NeuN 1:1000, MilliporeSigma catalog MAB377) at 4°C overnight. The next day, cells were incubated with fluorescently tagged secondary antibodies (anti-mouse Alexa Fluor 594, anti-rabbit Alexa Fluor 647, or anti-chicken Alexa Fluor 488, all at 1:500). Coverslips were mounted and sealed with Fluoromount media (Southern Biotech). The Nikon A1Rsi confocal microscope was used for acquiring images. GFAP intensity, branch length, and neuronal numbers were quantified using ImageJ.

### Quantitative real-time PCR.

RNA was isolated from mouse brain tissue using a RNeasy mini kit (Qiagen) according to the manufacturer’s protocol. Primary astrocytes were lysed using 500 μL of QIAzol Lysis Reagent (Qiagen) and placed into Eppendorf tubes. Chloroform (100 μL) was added to each sample, shaken vigorously for 5 seconds, and allowed to sit for 3 minutes at room temperature. The samples were centrifuged for 15 minutes at 12,000*g* at 4°C. The aqueous layer was removed and combined with 1.5 times volume of 100% ethanol and together added to the RNeasy column for RNA purification according to the manufacturer’s protocol. cDNA was generated using the High-Capacity cDNA Reverse Transcription Kit (Invitrogen) and analyzed on the QuantStudio 12K Flex Real-Time PCR System using either the Power SYBR Green PCR Master Mix (Thermo Fisher Scientific) or the PrimeTime Gene Expression Master Mix (Integrated DNA Technologies). Expression levels were calculated by the ΔΔCt method, normalized to GAPDH and a biological reference sample. Primer sequences or identifiers (purchased from Integrated DNA Technologies) are detailed in Supplemental Table 1.

### Glutamate uptake assay.

Glutamate uptake was assessed using the Glutamate Assay Kit (Abcam) per manufacturer’s instructions. Briefly, primary mouse astrocyte cultures and iAstrocytes were plated onto PDL-coated 24-well plates and treated with ASOs as described above. Cells were incubated in 100 μM glutamate in Live Cell Imaging Solution (Thermo Fisher Scientific) for 2 hours at 37°C. Following the incubation, 50 μL of the glutamate-containing media was combined with 50 μL of the reaction buffer in a 96-well plate and incubated for 30 minutes at room temperature with shaking while protected from light. Sample absorbance was measured at 450 nm on a plate reader, and a standard curve of known glutamate concentrations was used to quantify the concentration of glutamate within each sample. Samples were run in triplicate and averaged for final analyses.

### Cytotoxicity assay.

LDH release was quantified using the Pierce LDH Cytotoxicity Assay Kit (Thermo Fisher Scientific) following manufacturer’s instructions. Briefly, 24 hours prior to the assay, astrocyte medium was supplemented with 0 or 100 μM of hydrogen peroxide and added to the cells. The next day, the medium was collected, and LDH activity was measured at 490 nm and 680 nm on a plate reader. The 680 nm value was subtracted from the 490 nm value to obtain the level of LDH release. Samples were run in triplicate and averaged for final analyses.

### Microelectrode assay.

CytoView MEA Plates (Axion Biosystems) were coated with 100 μg/mL PDL in borate buffer (100 mM boric acid, MilliporeSigma, and 75 mM NaCl, MilliporeSigma), washed in molecular grade sterile water, and dried prior to cell seeding. Mixed human cortical neurons (BrainXell) were seeded with iAstrocytes at a 5:1 ratio in seeding medium. On day 4, the seeding medium was supplemented with the day 4 supplement from BrainXell. On day 7, the medium was replaced with Supplement C Treatment medium for 2 hours and then changed to maintenance medium. Media compositions are listed in Supplemental Table 2. Half medium changes were performed every 3 days until the end of the experiment. On day in vitro 21, electrical activity of the cultures was measured on the Axion Biosystems Maestro 768D MEA system. The cultures were equilibrated on the machine for 15 minutes at 37°C and 5% CO_2_. Recordings were taken for 20 minutes and repeated 3 times for each plate, at a sampling rate of 12.5 kHz. Spikes were detected using a threshold set to 6 times the estimated standard deviation of the noise. Single-channel bursts were detected as a minimum of 5 spikes and maximum 100 ms interspike intervals. Network bursts were detected as a minimum of 50 spikes, maximum 100 ms interspike intervals, and minimum of 35% participating electrodes. Analysis of individual spikes and bursts was done using the neural metrics tool from Axion Biosystems.

### Statistics.

Data were graphed as mean ± SEM and analyzed using GraphPad Prism 9 statistical software (GraphPad Software). For all experiments, male and female animals were combined and analyzed together. *P* < 0.05 was considered significant for all experiments.

### Study approval.

All husbandry and surgical procedures were approved by the Washington University Institutional Animal Care and Use Committee in accordance with federal standards.

## Author contributions

LAE, KMS, and TMM designed the experiments. LAE, KMS, CS, KH, and RM performed the experiments. LAE, CS, and KH analyzed the data. FR provided ASOs and advice regarding ASO use. MB, CMK, and RJB provided key reagents. LAE wrote the manuscript. All authors made substantial contributions to subsequent versions of the manuscript and approved of the final version.

## Supplementary Material

Supplemental data

## Version 1 12/07/2021

In-Press Preview

## Version 2 01/11/2022

Electronic publication
