# Supplementary material for: Astrocytic 4R tau expression drives astrocyte reactivity and dysfunction
Source: JCI Insight. 2022 Jan 11;7(1):e152012. doi: 10.1172/jci.insight.152012 (PMC8765054; doi:10.1172/jci.insight.152012)
Supplement: Supplemental data [file jciinsight-7-152012-s141.pdf]

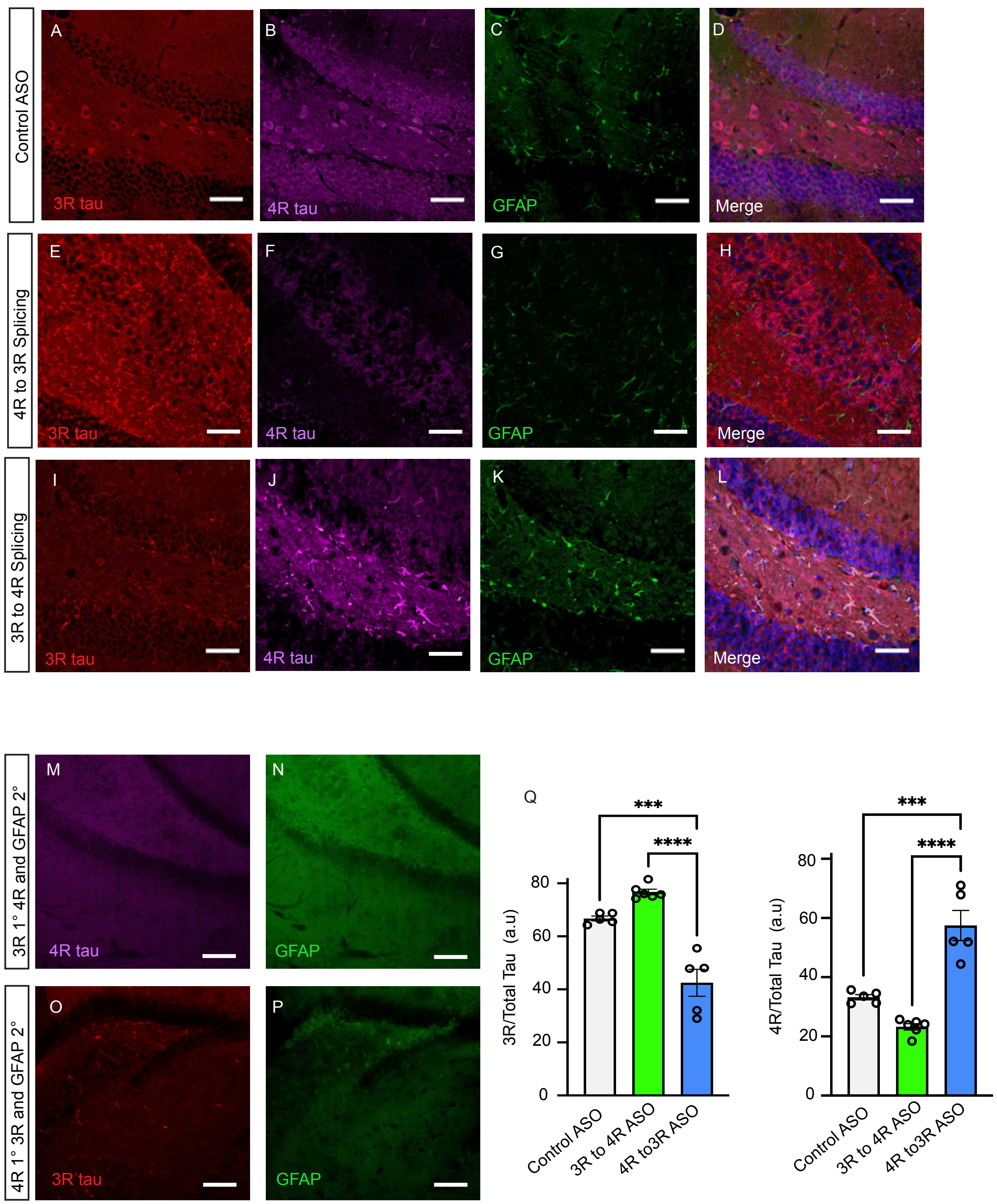

Supplementary Figure 1: Tau isoform localization *in vivo*. A, E and I) 3R tau, B, F, and J) 4R tau, C, G, and K) Gfap, D, H, and L) merged representative images of the dentate gyrus of the hippocampus, contralateral to ASO injection in hTau mice treated with control ASO or 3R to 4R tau splicing ASO. M and N) 4R and GFAP secondary staining in sections incubated with 3R tau primary antibody. O and P) 3R and GFAP secondary staining in sections incubated with 4R tau primary antibody. Scale bar = 50μm. Q) Relative amount of 3R and 4R tau compared to total tau. Data are shown as mean ± SEM n = 5-6 animals per treatment: ***p<0.001, ****p<0.0001,by one-way ANOVA with multiple corrections.

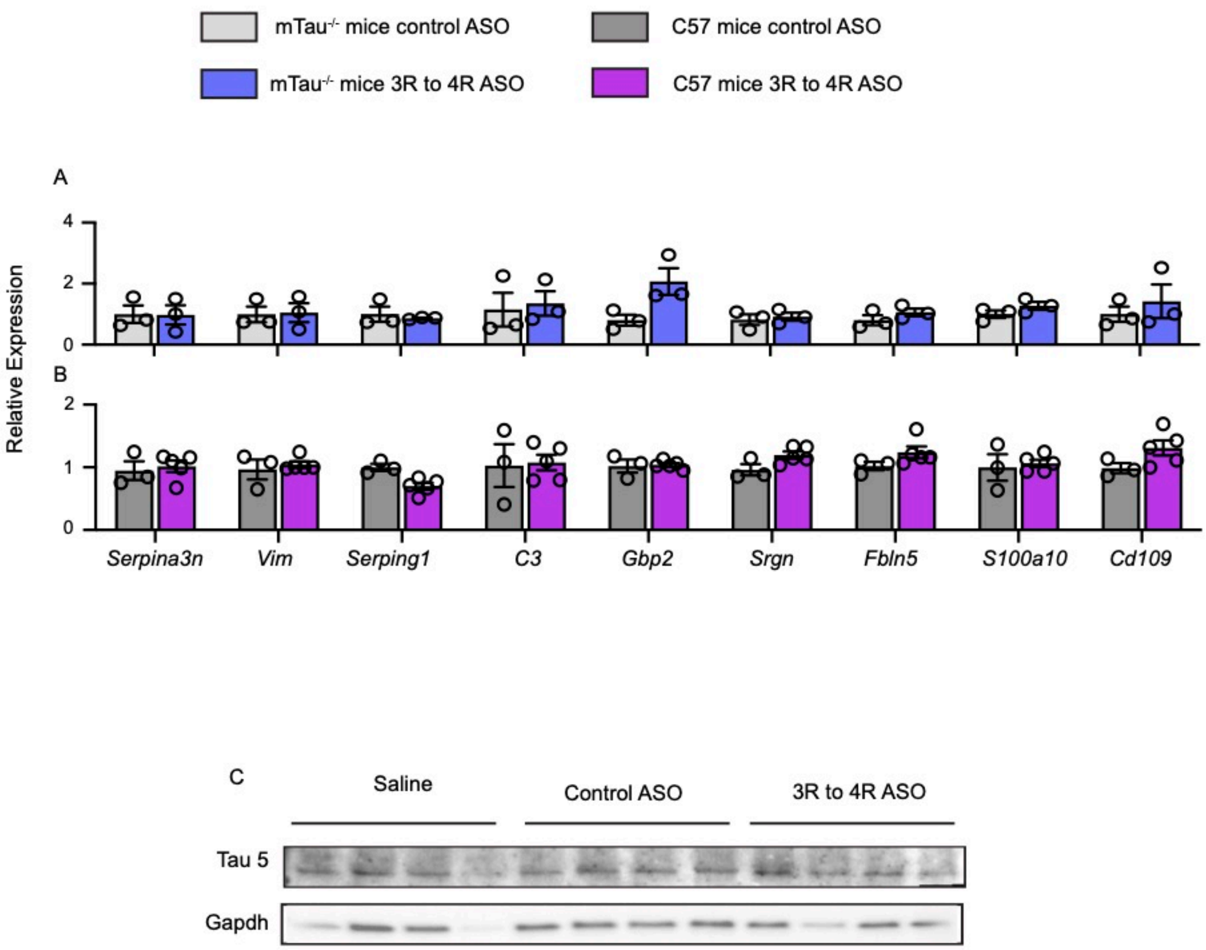

Supplementary Figure 2: ASO-mediated increase in 4R tau does not alter select mRNA levels in control mice. Expression of select pan-reactive (*Vimentin*, *Serpina3n*), neurotoxic (*Serping1*, *C3*, *Gbp2*, *Srgn* and *Fbln5*) and neuroprotective (*S100a10* and *Cd109*) genes in A) mTau-/- mice and B) C57BL6 (C57) mice were measured by qRT-PCR, normalized to *Gapdh*, and expressed relative to control ASO levels. Data are mean ± SEM. n=3-5 mice/group; two-way ANOVA multiple comparisons. C) Western blot for total tau of primary hTau astrocytes treated with saline, the control ASO, or 3R to 4R tau splicing ASO for 12 days, n=4 wells per condition.

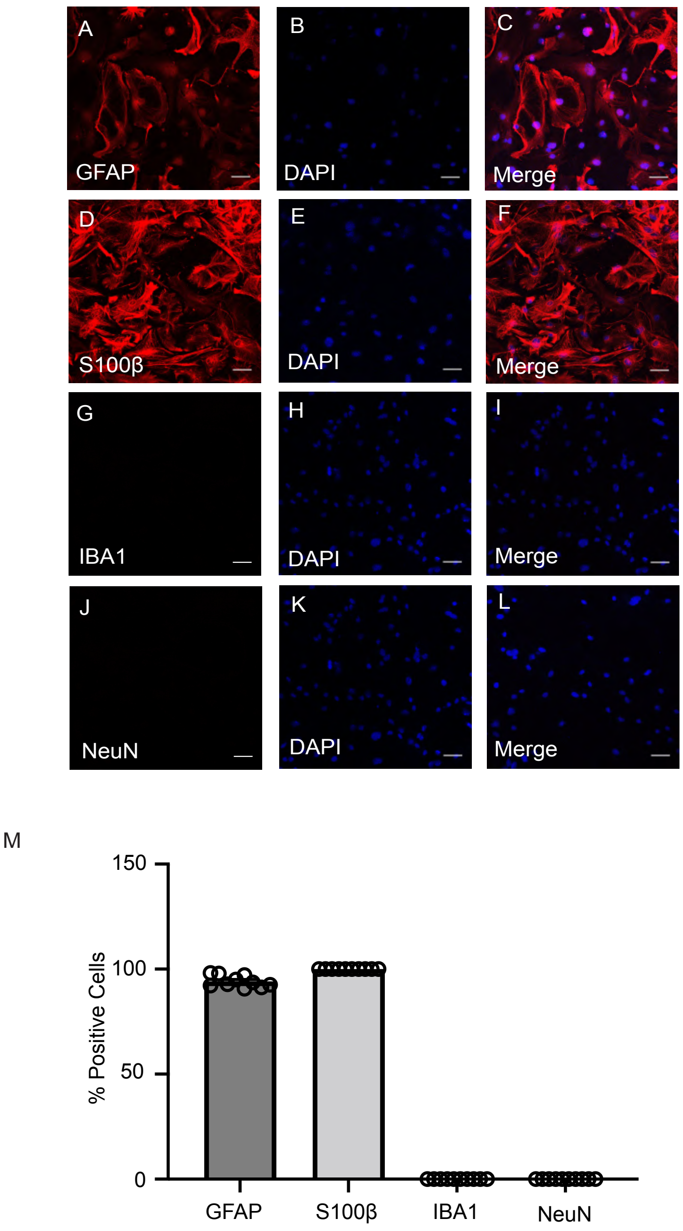

Supplementary Figure 3: Purity assessment of primary astrocyte cultures. Staining for A) GFAP, D) S100ß G) IBA1, J) NeuN, B, E, H, K) DAPI, and C, F, I, L) composite of respective images. M) Quantification of the number of positive cells for GFAP, S100ß, IBA1, and NeuN versus the total number of cells. Data are mean ± SEM; n=10 images per well.

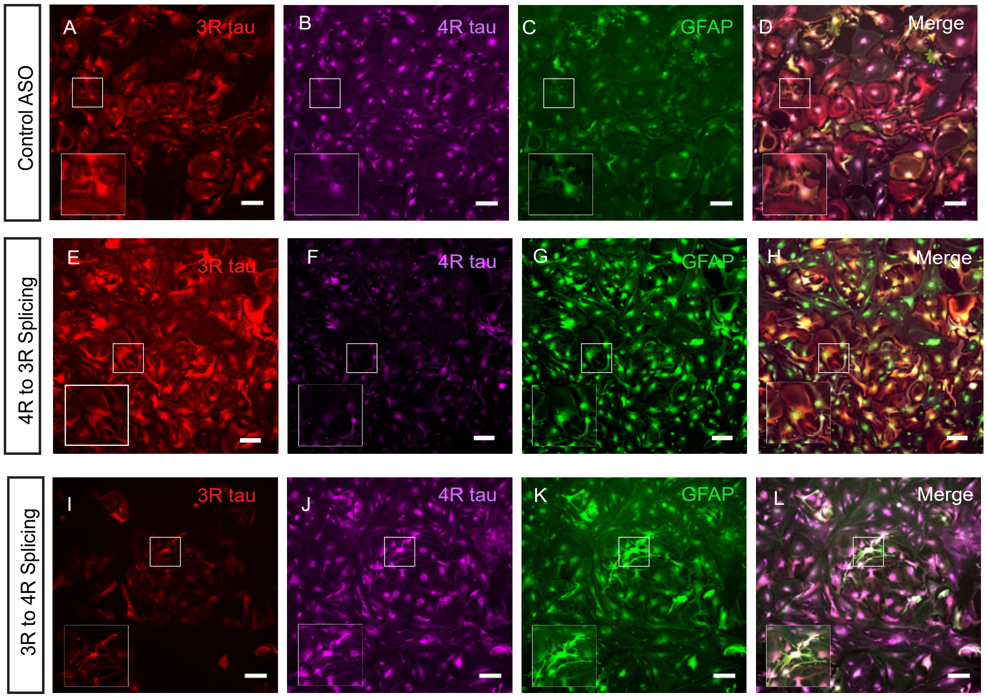

Supplementary Figure 4: Tau isoform localization in vitro. A, E and I) 3R tau, B, F, and J) 4R tau, C, G, and K) GFAP, and D, H, and L) merged representative images in primary hTau astrocytes treated with control ASO, 4R to 3R tau splicing ASO, or 3R to 4R tau splicing ASO. Scale bar = 200µM.

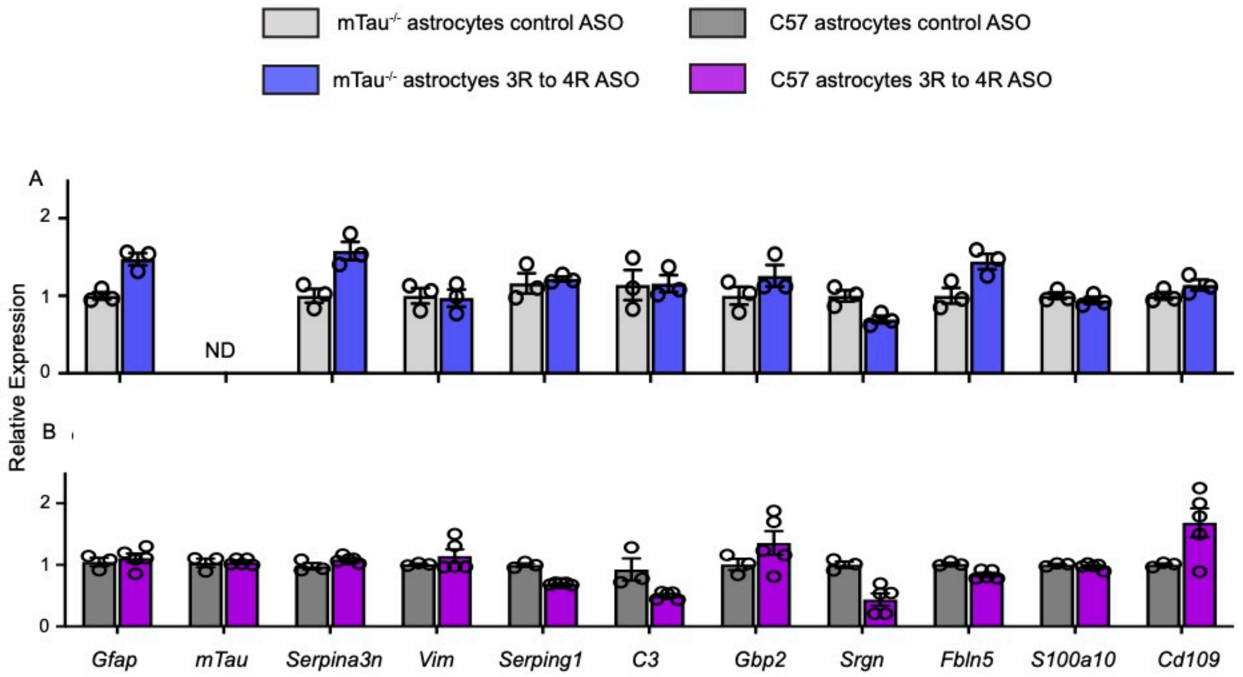

Supplementary Figure 5: ASO-mediated increase in 4R tau does not alter select mRNA levels in primary astrocyte cultures. Expression of select pan-reactive (*GFAP*, *Serpina3n, Vimentin*), neurotoxic (*Serping1*, *C3*, *Gbp2*, *Srgn* and *Fbln5*) and neuroprotective (*S100a10* and *Cd109*) genes in A) mTau-/- astrocytes and B) C57BL6 (C57) astrocytes were measured by qRT-PCR, normalized to *Gapdh*, and expressed relative to control ASO levels. Data are mean ± SEM; n=3-5 biological replicates/treatment; two-way ANOVA multiple comparisons. ND = not determined (cycle threshold values greater than 35).

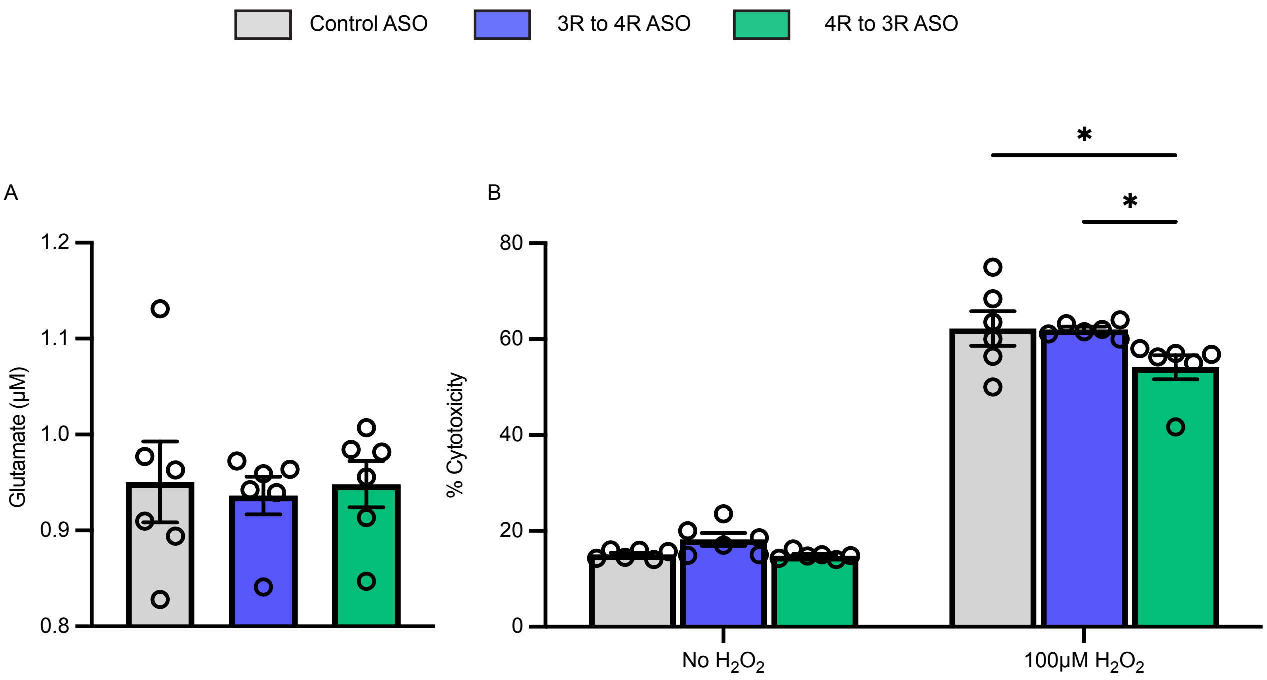

Supplementary Figure 6: Glutamate uptake and cytotoxicity in mTau-/- astrocytes following ASO treatment. A) Glutamate concentration measured in cellular media after control, 3R to 4R tau splicing, or 4R to 3R tau splicing ASO treatment in mTau-/- astrocytes. Data are mean ± SEM; n=6 biological replicates/treatment; one-way ANOVA with Tukey's multiple comparisons; ns = not significant. B) Cytotoxicity (measured by LDH release) in control, 3R to 4R tau splicing, or 4R to 3R splicing ASO treated mTau-/- astrocytes at baseline and following 100µM H_2O_2 treatment. Data are mean ± SEM; n=6 biological replicates/treatment; two-way ANOVA with Tukey's multiple comparisons; $*p<0.05$, ns = not significant.

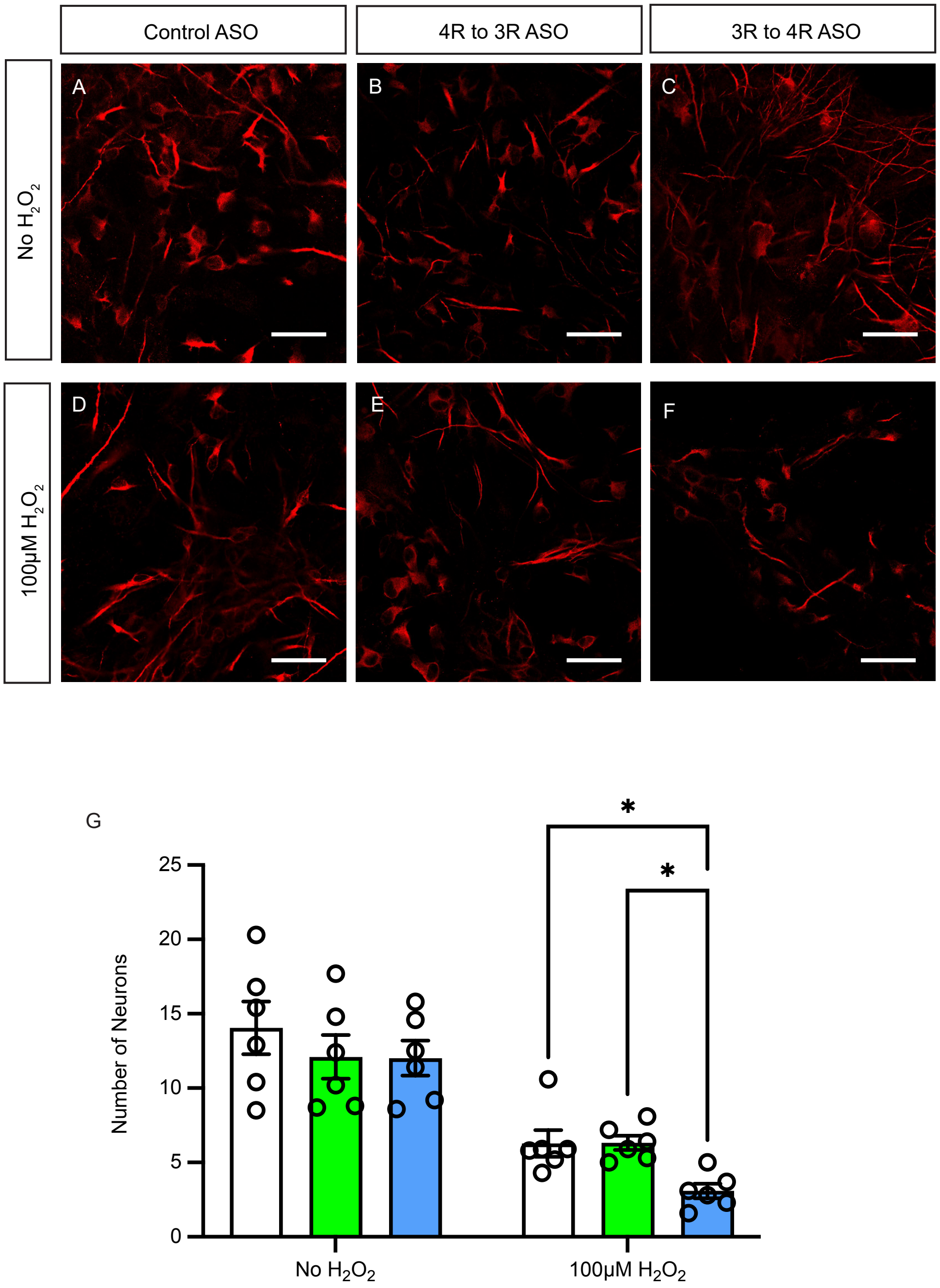

Supplementary Figure 7: Neurons cultured with 4R tau expressing astrocytes are more prone to death following oxidative stress. A-F) Representative images of MAP2 staining in iPSC cortical neurons co-cultured with hTau astrocytes treated with either control, 4R to 3R tau splicing or 3R to 4R tau splicing ASO at baseline and after hydrogen peroxide treatment. G) Quantification of the number of neurons in co-cultures. Data are shown as mean ± SEM, n= 6 wells per treatment and 10 images per well; *$p<0.05$, by two-way ANOVA with multiple corrections.

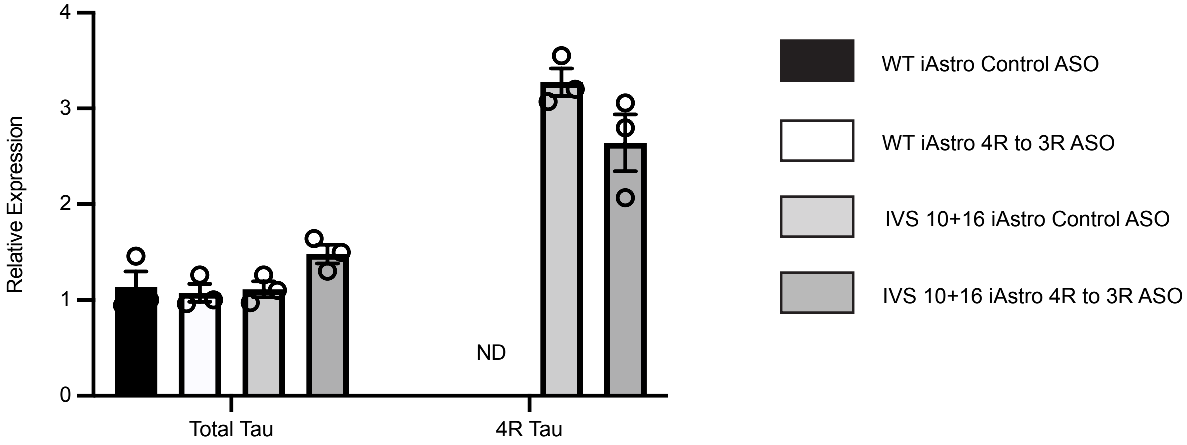

Supplementary Figure 8: Previously developed version of 4R to 3R tau splicing ASO does not efficiently alter mRNA levels of 4R tau in IVS 10+16 iAstrocytes. qRT-PCR data showing analysis of wild type (WT) and IVS 10+16 iAstrocytes treated with either a control ASO or a 4R to 3R tau splicing ASO (previously developed version). The IVS 10+16 mutation is located at the exact binding position of this version of ASO and does not effectively alter 4R tau mRNA levels. Data shown are mean ± SEM; n= 3 wells per treatment. ND = not determined (cycle threshold values greater than 35).

Gene Name	Species	Reagents	Sequence (5' to 3') or Assay ID
Serpina3n	M	Sybr	F: CAGATCCCAGCCATCAAGAG
			R: CTGGCAGCTGGCTGGTTT
S100a10	M	Taqman	Mm.PT.58.6571055 NM 009112
Cd109	M	Taqman	Mm.PT.58.6710335 NM 153098
Emp1	M	Taqman	Mm.PT.58.5886962 NM 010128
4R *MAPT* (4R Tau)	H	Sybr	F: GACTGGACGTTGCTAAGATC
			R: CATGCCAGACCTGAAGAATG
4R *MAPT* (4R Tau) Probe	H	Taqman	56-FAM/CCACTGAGAACCTGAAGCACCAGC/3IABkFQ
Total *MAPT* (Total Tau)	H	Sybr	F: AGAAGCAGGCATTGGAGAC
			R: TCTTCGTTTTACCATCAGCC
Total *MAPT* (Total Tau) Probe	H	Taqman	56-FAM/ACGGGACTGGAAGCGATGACAAAA/3IABkFQ
Gapdh	M	Sybr	F: TGCCCCCATGTTGTGATG
			R: TGTGGTCATGAGCCCTTCC
Gapdh Probe	M	Taqman	56-FAM/AATGCATCCTGCACCACCAACTGCTT/3IABkFQ
Gfap	M	Sybr	F: ACCGCATCACCATTCCTGTAC
			R: TGGCCTTCTGACACGGATTT
Gfap Probe	M	Taqman	56-FAM/TCCAGATCCGAGAAACCAGCCT/3IABkFQ
Fbln5	M	Taqman	Mm. PT.58.29865771 NM 011812
Serping1	M	Taqman	Mm. PT.58.30811631 NM 009776
Srgn	M	Taqman	Mm. PT.58.41483771 NM 011157
C3	M	Taqman	Mm. PT.58.17325540 NM 009778
Timp1	M	Sybr	F: AAGGTGGTCTCGTTGATTCG
			R: ATCTGGCATCCTCTTGTTGC
Vim	M	Sybr	F: TCCACTTTCCGTTCAAGGTC
			R: AGAGAGAGGAAGCCGAAAGC
Mapt (mTau)	M	Sybr	F: GAACCACCAAAATCCGGAGA
			R: CTCTTACTAGCTGATGGTGAC
Mapt (mTau) Probe	M	Taqman	56-FAM/CCAAGAAGGTGGCAGTGGTCC/3IABkFQ
GAPDH	H	Taqman	Hs.PT.39a.22214836 NM 002046(1)
GAPDH	H	Sybr	F: AGGGCTGCTTTTAACTCTGGT
			R: CCCCACTTGATTTTGGAGGGA
VIM	H	Sybr	F: TGGACCAGCTAACCAACGAC
			R: GCCAGAGACGCATTGTCAAC
SERPINA3	H	Sybr	F: CCTGAAGGCCCCTGATAAGAA
			R: GCTGGACTGATTGAGGGTGC
C3	H	Taqman	Hs.PT.56a.2840009 NM 000064(1)
SRGN	H	Sybr	F: AGGTTATCCTACGCGGAGAG
			R: GTCTTTGGAAAAAGGTCAGTCCT
GBP2	H	Sybr	F: CTATCTGCAATTACGCAGCCT
			R: TGTTCTGGCTTCTTGGGATGA
SERPING1	H	Sybr	F: CTGGCTGGGGATAGAGCCT
			R: GAGATAACTGTTGTTGCGACCT
FBLN5	H	Sybr	F: CTCACTGTTACCATTCTGGCTC
			R: GACTGGCGATCCAGGTCAAAG
S100A10	H	Sybr	F: GGCTACTTAACAAAGGAGGACC
			R: GAGGCCCGCAATTAGGGAAA
EMP1	H	Sybr	F: GTGCTGGCTGTGCATTCTTG
			R: CCGTGGTGATACTGCGTTCC
GFAP	H	Sybr	F: GTCCCCCACCTAGTTTGCAG
			R: TAGTCGTTGGCTTCGTGCTT

Supplementary Table 1: Sequences of primers used. Names and sequences of all primers and reagents used for qRT-PCR analysis. F, forward; R, reverse; M, Mouse; H, Human.

Seeding Media	Stock Concentration	Final concentration
DMEM/F12 (Life Technologies #11330-032)	1X	.5X
Neurobasal (Life Technologies #21103049)	1X	.5X
B27 (Life Technologies #17504-044)	50X	1X
N2 (Life Technologies #17502-048)	100X	1X
Glutamax (Life Technologies #35050-061)	200mM	.5mM
BDNF (Peprotech #450-02)	10ug/mL	10ng/mL
GDNF (Peprotech #450-10)	10ug/mL	10ng/mL
TGF-B1 (Peprotech #100-21C)	1ug/mL	1ng/mL
Seeding supplement (BrainXell, Madison, WI)	1000X	1X
Astrocyte supplement (BrainXell)	1000X	1X

Day 4 Media	Stock Concentration	Final concentration
DMEM/F12	1X	.25X
Neurobasal	1X	.25X
BrainPhys Media (STEMCELL Technologies #05790)	1X	.5X
B27	50X	1X
N2	100X	1X
Glutamax	200mM	.5mM
BDNF	10ug/mL	10ng/mL
GDNF	10ug/mL	10ng/mL
TGF-B1	1ug/mL	1ng/mL
Astrocyte supplement	1000X	1X
Day 4 supplement (BrainXell)	1000X	1X

Day 7 Supplement C Treatment	Stock Concentration	Final concentration
BrainPhys Media	1X	.5X
B27	50X	1X
N2	100X	1X
Glutamax	200mM	.5mM
Supplement C (BrainXell)	2000X	1X

Maintenance Media	Stock Concentration	Final concentration
BrainPhys Media	1X	.5X
B27	50X	1X
N2	100X	1X
Glutamax	200mM	.5mM
BDNF	10ug/mL	10ng/mL
GDNF	10ug/mL	10ng/mL
TGF-B1	1ug/mL	1ng/mL

Supplementary Table 2: Media composition for MEA assay. Components and concentration of reagents used for media in the MEA assay.

Seeding Media	Stock Concentration	Final concentration
DMEM/F12 (Life Technologies #11330-032)	1X	.5X
Neurobasal (Life Technologies #21103049)	1X	.5X
B27 (Life Technologies #17504-044)	50X	1X
N2 (Life Technologies #17502-048)	100X	1X
Glutamax (Life Technologies #35050-061)	200mM	.5mM
BDNF (Peprotech #450-02)	10ug/mL	10ng/mL
GDNF (Peprotech #450-10)	10ug/mL	10ng/mL
TGF-B1 (Peprotech #100-21C)	1ug/mL	1ng/mL
Seeding supplement (BrainXell, Madison, WI)	1000X	1X
Astrocyte supplement (BrainXell)	1000X	1X
**Day 1 Media**	**Stock Concentration**	**Final concentration**
DMEM/F12	1X	.25X
Neurobasal	1X	.25X
B27	50X	1X
N2	100X	1X
Glutamax	200mM	.5mM
BDNF	10ug/mL	10ng/mL
GDNF	10ug/mL	10ng/mL
TGF-B1	1ug/mL	1ng/mL
Astrocyte supplement	1000X	1X
Seeding supplement	1000X	1X
Geltrex (ThermoFisher Scientific #A1569601)	15mg/mL	15ug/mL
**Day 4 Media**	**Stock Concentration**	**Final concentration**
DMEM/F12	1X	.25X
Neurobasal	1X	.25X
B27	50X	1X
N2	100X	1X
Glutamax	200mM	.5mM
BDNF	10ug/mL	10ng/mL
GDNF	10ug/mL	10ng/mL
TGF-B1	1ug/mL	1ng/mL
Day 4 Supplement (BrainXell)	1000X	1X
Astrocyte supplement	1000X	1X
**Day 7 and on Media**	**Stock Concentration**	**Final concentration**
DMEM/F12	1X	.25X
Neurobasal	1X	.25X
B27	50X	1X
N2	100X	1X
Glutamax	200mM	.5mM
BDNF	10ug/mL	10ng/mL
GDNF	10ug/mL	10ng/mL
TGF-B1	1ug/mL	1ng/mL

Supplementary Table 3: Media composition for the iPSC-derived neuron and astrocyte co-cultures. Components and concentration of reagents used for media in the iPSC cortical neurons and iPSC-derived astrocytes.
